# Antibody validation and scoring guidelines for ABCG2 immunohistochemical staining in formalin-fixed paraffin-embedded colon cancer tissue

**DOI:** 10.1038/srep26997

**Published:** 2016-06-03

**Authors:** Camilla Natasha Cederbye, Jesper Andreas Palshof, Tine Plato Hansen, Anne Katrine Duun-Henriksen, Dorte Linnemann, Jan Stenvang, Dorte Lisbet Nielsen, Nils Brünner, Birgitte Martine Viuff

**Affiliations:** 1 Faculty of Health and Medical Sciences, Department of Veterinary Disease Biology, Section for Molecular Disease Biology, University of Copenhagen, Strandboulevarden 49, DK- 2100 Copenhagen Ø, Denmark; 2 Department of Oncology, Herlev and Gentofte Hospital, University of Copenhagen, Herlev Ringvej 75, DK-2730 Herlev, Denmark; 3 Department of Pathology, Hvidovre Hospital, Kettegaard Alle 30, DK- 2650 Hvidovre, Denmark; 4 Danish Cancer Society, Strandboulevarden 49, DK- 2100 Copenhagen Ø, Denmark; 5 Department of Pathology, Herlev and Gentofte Hospital, University of Copenhagen, Herlev Ringvej 75, DK-2730 Herlev, Denmark

## Abstract

Overexpression of the ATP-dependent drug efflux pump ABCG2 is a major molecular mechanism of multidrug resistance in cancer and might be a predictive biomarker for drug response. Contradictory results have been reported for immunohistochemical studies of ABCG2 protein expression in colorectal cancer (CRC), probably because of the use of different antibodies and scoring approaches. In this study, we systematically studied six commercially available anti-ABCG2 antibodies, using cell lines with up-regulation of ABCG2, and selected one antibody for validation in CRC tissue. Furthermore, we established scoring guidelines for ABCG2 expression based on the clinically used guidelines for HER2 immunohistochemistry assessment in gastric cancer. The guidelines provide a semi-quantitative measure of the basolateral membrane staining of ABCG2 and disregard the apical membrane staining and the cytoplasmic signal. Intra-tumor heterogeneity in ABCG2 immunoreactivity was observed; however, statistical analyses of tissue microarrays (TMAs) and the corresponding whole sections from primary tumors of 57 metastatic CRC patients revealed a strong positive correlation between maximum TMA scores and whole sections, especially when more than one core was used. In conclusion, here, we provide validated results to guide future studies on the associations between ABCG2 immunoreactivity in tumor cells and the benefits of chemotherapeutic treatment in patients with CRC.

Multidrug resistance (MDR) is defined as resistance to various chemotherapeutics that are diverse in both structure and function, and it is a major obstacle in cancer treatment. MDR may be pre-existing or acquired and can involve various cellular mechanisms, which frequently include the up-regulation of ATP-binding cassette transporters (ABC transporters). ABC transporters are encoded by 48 different genes, which are divided into 7 subfamilies (A–G). ABCG2, also known as breast cancer resistant protein (BCRP), is encoded by the *ABCG2* gene and belongs to the G subfamily of ABC transporters^1^. It was first discovered in the multidrug resistant breast cancer cell line MCF7/AdrVp^2^ and has since been found in both tumor tissue and normal tissue^3^,^4^. In contrast to most ABC transporters, ABCG2 is a half transporter and is functionally active only as a dimer or multimer^5^. Substrates for ABCG2 include chemotherapeutic drugs such as mitoxantrone, doxorubicin, 5-fluorouracil (5FU) and SN-38 (the active metabolite of irinotecan)^6^. Several studies have suggested an association between ABCG2 up-regulation in hematologic malignancies and solid tumors and prognosis/efficacy of treatment^7^,^8^, but the value of ABCG2 as a clinically validated biomarker has yet to be established. The treatment of colorectal cancer (CRC) includes surgery and combination therapy regimes containing ABCG2 substrates such as 5FU and irinotecan. On the basis of what is currently known, these drugs are expected to have little or no effect in cancer patients with ABCG2 up-regulation in the cancer cells. Thus, ABCG2 may function as a predictive biomarker of the efficacy of chemotherapeutic treatment. However, validation and clinical implementation of ABCG2 as a biomarker in the clinic greatly depends on a reliable and validated detection method for the *ABCG2* gene, mRNA or protein. The relationship between ABCG2 expression and patient outcome and chemotherapy resistance has not been established, owing to conflicting results^9^. Previous immunohistochemical studies of the association between ABCG2 protein expression and clinical outcome have used different antibodies and different scoring guidelines^10^,^11^,^12^,^13^. At present, no universally accepted guidelines exist for the analytical or clinical validation of ABCG2 in clinical tumor tissues.

The present study focused on validation of anti-ABCG2 antibodies for the detection of ABCG2 protein expression in formalin-fixed paraffin-embedded (FFPE) CRC tissue samples. Six commercially available anti-ABCG2 antibodies were validated using three different SN-38 resistant cell lines with drug-induced up-regulation of ABCG2 along with their parental counterparts. Based on the obtained results, we selected one antibody that exhibited high sensitivity, specificity and reproducibility. Here, we provide new immunohistochemical scoring guidelines for ABCG2 based on the scoring guidelines for HER2, which has been successfully applied in clinical settings. These guidelines were used to investigate the correlation between ABCG2 basolateral membrane staining in TMA and whole sections of CRC tissue.

## Results

The specificity of six commercially available anti-ABCG2 antibodies was evaluated by western blotting (WB) and immunocytochemistry (ICC) assays using the LoVo, MDA-MB-231, and MCF7 cell lines, each with an ABCG2 up-regulated variant (Table 1). The validation and selection protocols are visualized in Supplementary Figure S1.

### Antibody validation by western blotting

**Figure 1:**
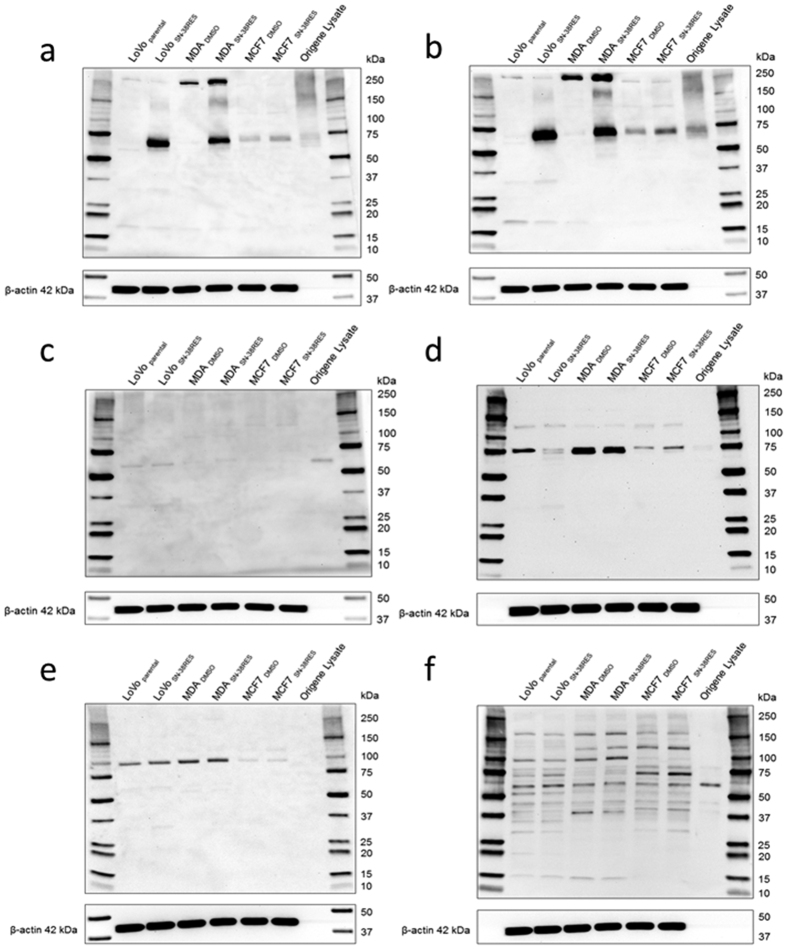
Western blot evaluation of the specificity of six commercially available anti-ABCG2 antibodies. The full membrane is displayed to reveal the presence of all reactive bands upon antibody incubation. The six different antibodies were applied to ABCG2 up-regulated (SN-38 RES) and parental LoVo, MDA-MB-231 and MCF7 cancer cell lines as indicated in the figure. The six different antibodies are applied in the six panels as follows: (**a**) mAb BXP-21, (**b**) mAb 6D171, (**c**) mAb 3G8, (**d**) pAb B7185, (**e**) pAb TA332085 and (**f**) pAb TA324234. β-actin (42 kDa) was used as a loading control. The molecular weight marker is indicated.

The mouse monoclonal antibody (mAb) BXP-21 reacted with a 72 kDa protein corresponding to ABCG2 in LoVo_SN-38RES_ and MDA_SN-38RES_. As expected, no 72 kDa bands were observed for the corresponding parental cell lines LoVo_parental_ and MDA_DMSO_ (Fig. 1a). A 72 kDa band was observed for both MCF7_SN-38RES_ and MCF7_DMSO_; however, the band in MCF7_SN-38RES_ was slightly more intense than the band in MCF7_DMSO_. Furthermore, a weak 140–150 kDa band was observed for LoVo_SN-38RES_ and MDA_SN-38RES_ but not for the parental cell lines. At approximately 250 kDa, intense bands were detected in MDA_DMSO_ and MDA_SN-38RES_. Additionally, barely visible bands between 15 kDa and 20 kDa were observed in both LoVo _SN-38RES_ and LoVo_parental_. For the transient ABCG2-overexpressing Origene lysate, only a blurred lane displaying an indistinct band at approximately 72 kDa was observed. Only 2 μg of the protein lysate from overexpressing cells was loaded, and the concentration was too low for detection of the 42 kDa loading control, β-actin. The results were verified five times by WB for LoVo cells, but the Origene transient overexpression lysate was included only once.

The western blot results for the mouse mAb 6D171 were very similar to those for BXP-21 (Fig. 1b). Mouse mAb 6D171 detected intense bands at 72 kDa corresponding to ABCG2 in LoVo_SN-38RES_ and MDA_SN-38RES_ and, as expected, not in LoVo_parental_ and MDA_DMSO_. Weaker bands of the same size were seen in MCF7_DMSO_, and somewhat more intense bands of the same size were observed in MCF7_SN-38RES_. Additional bands were observed at 250 kDa in MDA_SN-38RES_ and MDA_DMSO_, faint bands were observed at 140–150 kDa in LoVo_SN-38RES_ and MDA_SN-38RES_, and faint bands were observed at 15–20 kDa in LoVo_parental_ and LoVo_SN-38RES_. The results for 6D171 were verified five times by WB for LoVo cells, and the Origene transient overexpression lysate was included once.

The mouse mAb 3G8 detected faint bands at approximately 60 kDa in LoVo_SN-38RES_, LoVo_parental_, MDA_DMSO_ and MDA_SN-38RES_ cell lines, whereas no bands were detected at 72 kDa (Fig. 1c). The most intense band was detected in the ABCG2-overexpressing lysate from Origene, also at approximately 60 kDa. No bands were observed for MCF7_DMSO_ and MCF7_SN-38RES_. The results for 3G8 were tested four times by WB for LoVo cells, and the Origene overexpression lysate was included once. In our hands, it was not possible to optimize the results for 3G8 with antibody titration and different blocking solutions (data not shown).

The rabbit polyclonal antibody (pAb) B7185 detected a band at 75 kDa in all cell lines (Fig. 1d). For LoVo_SN-38RES_, the 75 kDa band was weak compared to that for LoVo_parental_ but a second, slightly lower band was detected in LoVo_SN-38RES_, and the size of this band was close to the expected size of 72 kDa. As in LoVo_SN-38RES_, two close bands were observed in MCF7_SN-38RES_, whereas only the 75 kDa band was observed in the corresponding MCF7_DMSO_. Two very intense bands at 72–75 kDa were observed in both MDA_DMSO_ and MDA_SN-38RES_. In the Origene lysate supposedly overexpressing ABCG2, two barely visible bands at 75 kDa were observed. Faint bands were also seen at 125 kDa in all cell lines, both parental and ABCG2 up-regulated. The results for B7185 were verified five times by WB for LoVo cells, but the Origene overexpression lysate was included only once.

A distinct band at approximately 100 kDa was detected for all cell lines for the rabbit pAb TA332085 (Fig. 1e). There was no difference in intensity for this band in ABCG2 up-regulated cells and their parental counterparts. Additionally, barely visible bands were seen between 50–75 kDa and 25–37 kDa in LoVo_parental_ and LoVo_SN-38RES_. No band was observed in the Origene lysate. The specificity of TA332085 was tested by WB four times for LoVo cells, and the Origene overexpression lysate was included once.

The rabbit polyclonal antibody TA324234 detected multiple bands in both ABCG2 up-regulated and parental cell lines; however, no distinct band at 72 kDa was observed for any cell line (Fig. 1f). The size of the bands was different among the cell lines but no difference was seen between the ABCG2 up-regulated and corresponding parental cell lines. An intense band was detected in the ABCG2-overexpressing lysate from Origene at 60 kDa. The specificity of TA324234 was tested four times using both 5% skim milk and 5% BSA as blocking solutions, and different antibody concentrations were tested (data not shown).

Based on the WB results, the three antibodies 3G8, TA332085 and TA324234 were excluded from further investigation, even though it was possible that they would have worked in immunohistochemistry (IHC), because we used the band size determined by WB as a criterion to determine the specificity of the antibodies. The three antibodies BXP-21, 6D171 and B7185 were selected for further validation by ICC using ABCG2 up-regulated cell lines and parental counterparts.

### Antibody validation by ICC

**Figure 2:**
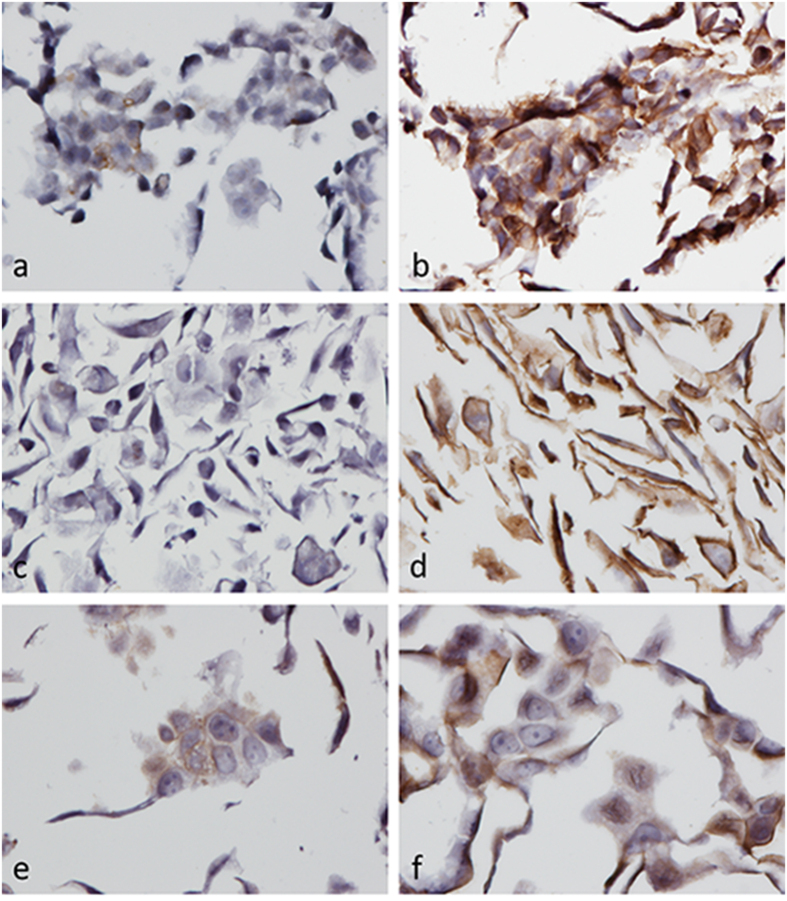
Immunostaining of ABCG2 using mAb BXP-21 (1:3000 dilution) on LoVo, MDA-MB-231, and MCF7 parental and SN-38 resistant cell lines counterstained with Mayer’s hematoxylin. ABCG2 was visualized with DAB (brown), 40x magnification. (**a**) LoVo_parental_ (**b**) LoVo_SN-38RES_ (**c**) MDA_DMSO_ (**d**) MDA_SN-38RES_ (**e**) MCF7_DMSO_ and (**f**) MCF7_SN-38RES_.

A clear difference in ABCG2 immunostaining was observed between the ABCG2 up-regulated cell lines and their corresponding parental cell lines when using BXP-21 for ICC. Weak ABCG2 immunostaining was observed in the cytoplasm of a small fraction of LoVo_parental_ cells (Fig. 2a), whereas the ABCG2 immunostaining observed in LoVo_SN-38RES_ cells included most cells and was observed as a very strong staining of both the membrane and cytoplasm (Fig 2b). No ABCG2 immunostaining was observed in MDA_DMSO_ cells (Fig. 2c), whereas very strong membrane staining was observed in MDA_SN-38RES_ cells (Fig. 2d). As expected, BXP-21 detected ABCG2 in both MCF7_DMSO_ and MCF7_SN-38RES_ cells, although the membrane staining was more visible for the MCF7_SN-38RES_ cells (Fig. 2e,f).

When using mAb 6D171 for ICC, the results were identical to the results described above for BXP-21. Again the ABCG2 immunostaining in the ABCG2 up-regulated cell lines was considerably stronger than the corresponding parental cell lines, and pronounced membrane staining was observed. No ABCG2 immunostaining was observed in MDA_DMSO_, whereas a weak signal was obtained in a subpopulation of LoVo_parental_ and MCF7_DMSO_ cells (Supplementary Figure S2).

Distinct membrane and diffuse cytoplasmic staining was observed in ABCG2 up-regulated cell lines as well as in their parental counterparts when the rabbit pAb B7185 was used for ICC (Supplementary Figure S3). The intensity of the ABCG2 immunostaining was similar for the parental and the ABCG2 up-regulated cells, and prominent membrane staining was observed in both MDA_DMSO_ and MDA_SN-38RES._

### Down-regulation of ABCG2 by RNA interference

LoVo_parental_, LoVo_SN-38RES_, MDA_DMSO_ and MDA _SN-38RES_ cell lines were transfected with a mixture of three different small interfering RNA (siRNA) targeting ABCG2 to verify antibody specificity. Transfection efficiency was evaluated by transfection with a fluorescent labeled siRNA and was investigated by fluorescence microscopy. This revealed that only a fraction of LoVo_SN-38RES_ cells were transfected.

Four *ABCG2* transcripts were found on ensembl.org, two of which are known protein coding transcripts and two of which are putative protein coding transcripts. Reference sequences are available for the protein coding variants, which comprise 4479 bp and 4276 bp, encoding 655aa and 611aa proteins, respectively. The 655aa protein has a predicted molecular weight of 72.7 kDa and the 611aa protein has a predicted molecular weight of 67.8 kDa, because 1 kb is equivalent to 37 kDa. The two transcripts differ only in exons 14 and 16. The applied siRNA complementary sequences in exons 7, 8, and 9, i.e., both of the protein coding splice variants, were targets of the siRNA.

**Figure 3:**
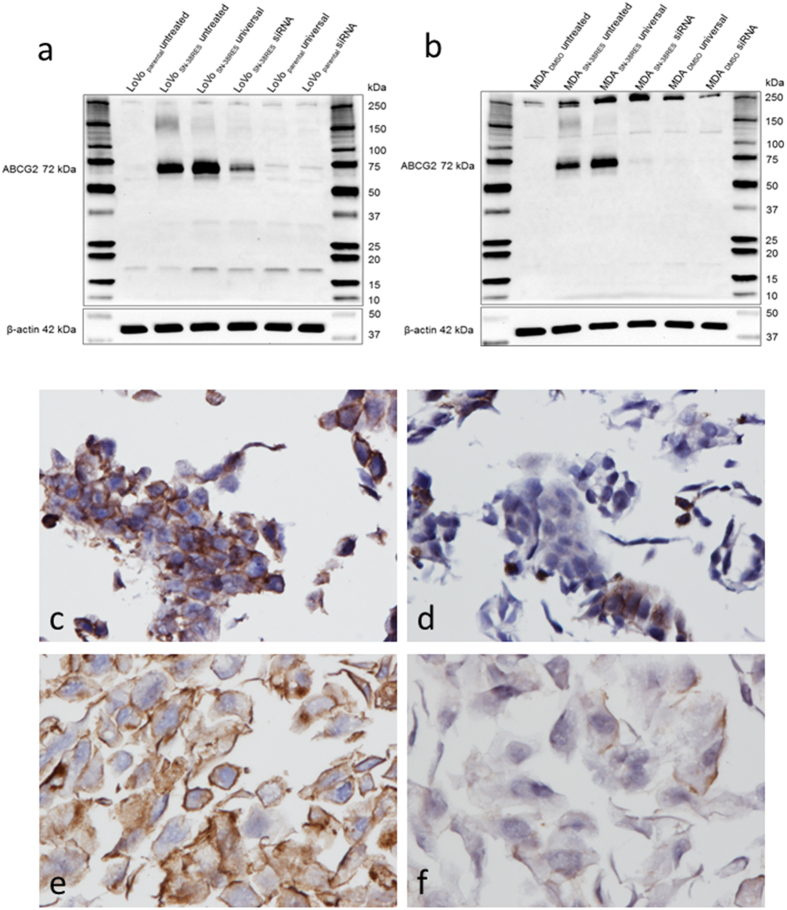
(**a,b**) Western blot evaluation of mAb BXP-21 specificity by siRNA-mediated down-regulation of ABCG2 (72 kDa). Two parental cell lines (LoVo_parental_ and MDA_DMSO_) were analyzed alongside their SN38 resistant counterparts (LoVo_SN-38RES_ and MDA_SN-38RES_) with up-regulated levels of ABCG2 as indicated above the membranes. (**a**) LoVo_parental_ and LoVo_SN-38RES_ and (**b**) MDA_DMSO_ and MDA_SN-38RES_. The cells were untreated, transfected with a universal negative control siRNA (universal) or transfected with a mixture of three different ABCG2-targeting siRNAs as indicated above the membranes. β-actin (42 kDa) was used as a loading control. The molecular weight marker is indicated. (**c–f**) Immunostaining of ABCG2 in siRNA down-regulated LoVo_SN-38RES_ and MDA_SN-38RES_ with BXP-21 (1:3000 dilution). ABCG2 was detected using the HiDef Detection^TM^ HRP Polymer system and counterstained with Mayer’s hematoxylin, 40x magnification. (**c**) LoVo_SN-38RES_ transfected with universal siRNA. (**d**) LoVo_SN-38RES_ transfected with ABCG2-specific siRNA. (**e**) MDA_SN-38RES_ transfected with universal siRNA. (**f**) MDA_SN-38RES_ transfected with ABCG2-specific siRNA. A partial ABCG2 down-regulation was observed in LoVo_SN-38RES_, whereas an almost complete down-regulation occurred in MDA SN-38 as evidenced by reduced immunostaining.

A substantial reduction in the 72 kDa band of ABCG2 was demonstrated with mAb BXP-21 in MDA_SN-38RES_ 96 hours after transfection and to a lesser extent in LoVo_SN-38RES_ (Fig. 3a,b). Furthermore, the faint 140–150 kDa band observed in the untreated LoVo_SN-38RES_ and MDA_SN-38RES_ disappeared completely in the ABCG2 siRNA down-regulated cell lines. The faint bands between 15 kDa and 20 kDa observed in both LoVo_parental_ and LoVo_SN-38RES_ were not affected by ABCG2-specific siRNA, nor were the intense bands at 250 kDa in MDA_DMSO_ and MDA_SN-38RES_. Additional siRNA-transfected cells, cultured in parallel with cells for WB, were fixed in formalin and paraffin embedded for the ICC assay. Consistently with the WB results, ICC showed that mAb BXP-21 demonstrated an almost complete down-regulation of ABCG2 in siRNA-transfected MDA_SN-38RES_ cells, whereas only a partial down-regulation was observed in the siRNA-transfected LoVo_SN-38RES_ cells. A distinctive ABCG2 staining was observed in the membrane of LoVo_SN-38RES_ and MDA_SN-38RES_ cells transfected with universal control siRNA (Fig. 3c–f).

Similarly, an almost complete knockdown of the 72 kDa band was demonstrated in MDA_SN-38RES_ and, to a lesser extent, in LoVo_SN-38RES_ when mAb 6D171 was subjected to WB analysis. No down-regulation of additional bands was observed for 6D171 (Supplementary Figure S4a,b) except for the 140–150 kDa band, for which complete knockdown was observed. Furthermore, an almost complete down-regulation of ABCG2 in siRNA-transfected MDA_SN-38RES_ cells and a partial down-regulation in siRNA-transfected LoVo_SN-38RES_ cells were demonstrated by ICC for mAb 6D171 (Supplementary Figure S4c–f).

**Figure 4:**
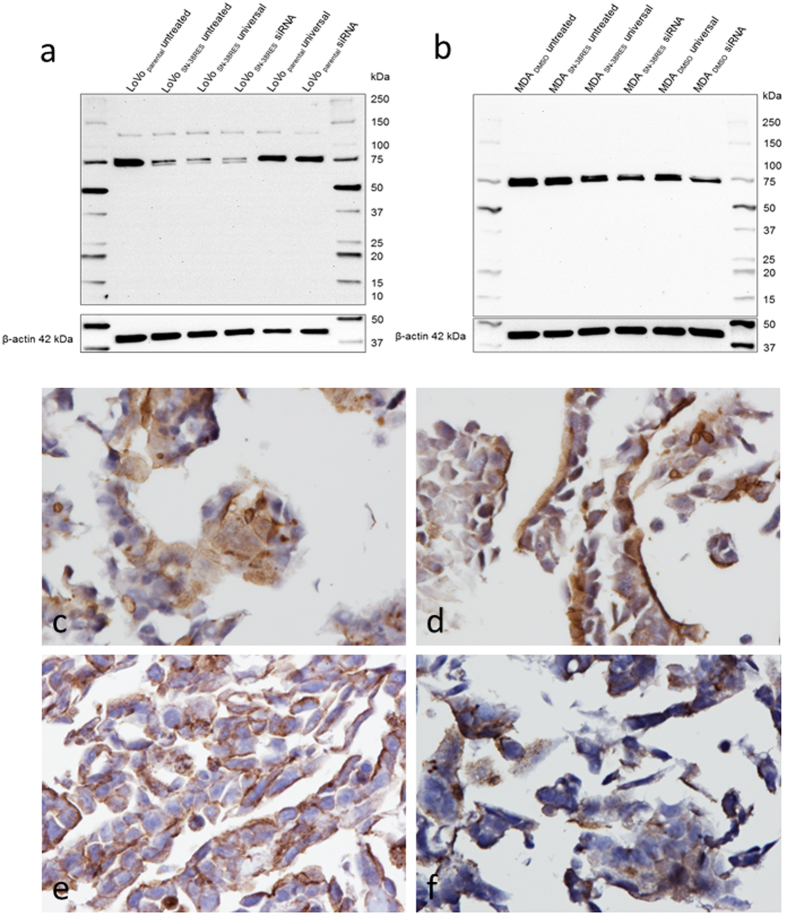
(**a,b**) Western blot evaluation of pAb B7185 specificity by siRNA-mediated down-regulation of ABCG2 (72 kDa). Two parental cell lines (LoVo_parental_ and MDA_DMSO_) were analyzed in comparison to their SN38 resistant counterparts (LoVo_SN-38RES_ and MDA_SN-38RES_) with up-regulated levels of ABCG2 as indicated above the membranes. (**a**) LoVo_parental_ and LoVo_SN-38RES_ and (**b**) MDA_DMSO_ and MDA_SN-38RES_. The cells were untreated, transfected with a universal negative control siRNA (universal) or transfected with a mixture of three different ABCG2-targeting siRNAs as indicated above the membranes. β-actin (42 kDa) was used as a loading control. The molecular weight marker is indicated. (**c–f**) Immunostaining of ABCG2 in siRNA down-regulated LoVo parental and LoVo_SN-38RES_ with B7185 (1:500 dilution). ABCG2 was detected using the HiDef Detection^TM^ HRP Polymer system and counterstained with Mayer’s hematoxylin, 40x magnification. (**c**) LoVo_parental_ transfected with universal siRNA. (**d**) LoVo_parental_ transfected with ABCG2-specific siRNA. (**e**) LoVo_SN-38RES_ transfected with universal siRNA. (**f**) LoVo_SN-38RES_ transfected with ABCG2-specific siRNA.

With the pAb B7185, two bands in proximity at 72–75 kDa were observed by WB for LoVo_SN-38RES_. After transfection with ABCG2-specific siRNA, both bands were still present; however, the upper band appeared faint compared to the intense band in the universal siRNA LoVo_SN-38RES_ control (Fig. 4a). Regarding MDA_SN-38RES_ and MDA_DMSO_, two similar bands were observed with B7185, although they were more difficult to distinguish from each other. The ABCG2-specific siRNA-transfected MDA_SN-38RES_ and MDA_DMSO_ both displayed weaker upper bands than the universal siRNA control (Fig. 4b). In contrast to the results obtained with mAbs BXP-21 and 6D171, there were no differences in the immunostaining for the ABCG2 siRNA-transfected cells and the universal siRNA-transfected cells when pAb B7185 was used for ICC (Fig. 4c–f).

### Antibody validation by IHC

A strong ABCG2 immunoreactivity was observed in the apical membrane of luminal epithelial cells in part of the colon epithelium (Supplementary Figure S5) and occasionally in the basolateral membrane of crypt epithelial cells when mAb BXP-21 was used for IHC analysis of FFPE normal colon tissue. In normal liver, the bile canaliculi demonstrated a strong immunostaining, and furthermore, immunostaining was seen in endothelial cells in part of the vessels in the submucosa in the colon and in the liver (Supplementary Figure S5). An identical result was obtained when mAb 6D171 was used for IHC on normal colon tissue and normal liver tissue. Likewise, when pAb B7185 was used for IHC, immunostaining of the apical membranes of luminal epithelial cells of the colon was demonstrated but, furthermore, strong immunostaining of the membrane and cytoplasm of mononuclear cells in the lamina propria was obtained (data not shown).

### Cross-reactivity toward ABCB1

Based on the results described above, BXP-21 was chosen for IHC analysis of clinical CRC tissue samples. To investigate possible cross-reactivity between ABCG2 and ABCB1 (144 kDa), which is another prominent ABC transporter associated with cancer cell drug resistance, the ABCB1 expression level in the SN38 resistant cell lines and in their parental counterparts were evaluated by WB (Supplementary Figure S6). No bands were detected at 144 kDa for MDA_SN-38RES_ and MCF7_SN-38RES_, whereas ABCB1 was up-regulated in LoVo_SN-38RES_. Therefore, to evaluate a possible cross-reactivity to ABCB1 for mAb BXP-21, MDA-MB-231 and MCF7 cells with very high ABCB1 expression, due to a docetaxel-induced up-regulation, and their parental counterparts were subjected to WB and ICC. The LoVo_parental_ and LoVo_SN-38RES_ cell lines were included for WB. Several faint bands were detected when BXP-21 was used for WB including a faint band at 140–150 kDa in LoVo_SN-38RES_ but not in the two ABCB1 up-regulated cell lines (Supplementary Figure S7a). Furthermore, a distinct band at 72 kDa was observed for MCF7_parental_ and a faint band at 72 kDa for MCF7_DTRES_, as MCF7 cells express low levels of ABCG2. No immunostaining was seen in FFPE docetaxel resistant MDA_parental_ by ICC, and only very weak, mainly cytoplasmic staining was detected in MDA_DTXRES_ (Supplementary Figure S7b,c). For both MCF7_parental_ and MCF7_DTXRES_, immunostaining of the membrane was demonstrated by ICC (Supplementary Figure S7d,e). However, the intensity of the immunostaining of the ABCB1 up-regulated cell line was the same as that of the parental counterpart. The potential cross-reactivity of BXP-21 was tested three times.

### Scoring guidelines

**Figure 5:**
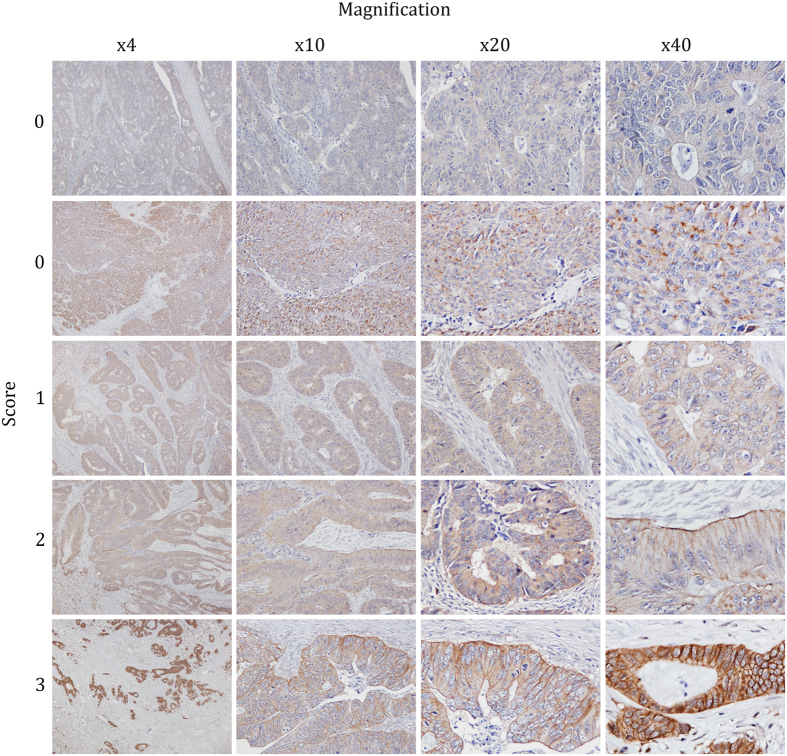
Immunostaining of ABCG2 in CRC tissue with mouse mAb BXP-21 (1:500 dilution). ABCG2 was detected using the HiDef Detection^TM^ HRP Polymer system and counterstained with Mayer’s hematoxylin. Row 1: No basolateral membrane staining is defined as 0. Row 2: No basolateral membrane staining but moderately strong cytoplasmic staining is defined as 0. Row 3: Weak basolateral staining visible only at 40x magnification is defined as 1. Row 4: Weak to moderate basolateral membrane staining visible at 10/20x magnification is defined as 2. Row 5: Strong basolateral membrane staining visible at 4x magnification is defined as 3.

Observations from IHC with mAb BXP-21 of the 9 FFPE CRC tissue samples revealed three distinguishable ABCG2 expression patterns of the tumor cells, namely basolateral membrane, apical membrane and cytoplasmic, which could be detected separately or in combination. As the functional ABCG2 transporter is a membrane protein, we decided to focus on the membrane staining and to apply the established HER2 IHC scoring guidelines on ABCG2 IHC to establish a standardized semi-quantitative method to measure ABCG2 in CRC tissue. The staining intensity of the basolateral membranes was evaluated and given a score between 0–3. An overview of the suggested guidelines is shown in Table 2. The score 0 was given if no basolateral membrane staining was observed or if basolateral membrane staining was observed in less than 10% of the tumor cells at 40x magnification (Fig. 5 row 1). Although cytoplasmic staining in tumor cells was observed for some patients, the score was considered to be 0 if no basolateral membrane staining was observed (Fig. 5 row 2). Weak basolateral membrane staining in ≥10% of the tumor cells visible only at 40x magnification, was scored as 1 (Fig. 5 row 3), whereas weak to moderate basolateral membrane staining in ≥10% of the tumor cells visible at 10/20x magnification was scored as 2 (Fig. 5 row 4). Finally, strong basolateral membrane staining in ≥10% of the tumor cells visible at 4x magnification was scored as 3 (Fig. 5 row 5).

### Effect of fixation

#### Cell lines

To investigate whether the duration of fixation affects the antibody-antigen reaction with BXP-21, LoVo_parental_ and LoVo_SN-38RES_ were fixed in 10% neutral buffered formalin (NBF) for 5 minutes, 30 minutes, 6 hours, 1 week, or 1 month prior to paraffin embedding. The intensity of ABCG2 immunoreaction appeared to be unaffected in cells fixed in formalin for 30 minutes, 6 hours, 1 week, or 1 month, whereas cells fixed for only 5 minutes had a slightly weaker immunoreaction compared to the other fixation times (Supplementary Figure S8).

#### Tissue biopsies

**Figure 6:**
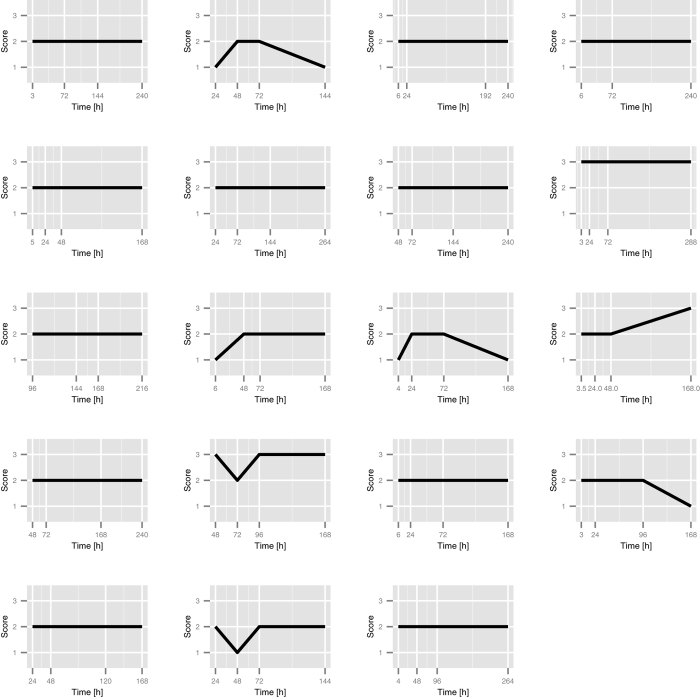
Effect of fixation times in tissues from 19 CRC patients. Each plot shows the score at each time point for a single patient.

The influence of fixation on the antigen-antibody reaction was further investigated with IHC on FFPE CRC tissues from 19 patients. Each tumor sample was divided into four smaller specimens that were fixed in NBF for 3 to 288 hours. Guiding durations of fixation were 6, 24, 48, and 168 hours; however, several samples deviated from these time points. The IHC assay was performed with mAb BXP-21, after which each sample was assigned a score from 0–3 for basolateral membrane staining. As shown in Fig. 6, changes in scores over time were observed in only a few patients. Because the scores were ordinal, a proportional odds model was used to analyze the effects of different fixation times (exposure) on basolateral membrane scores (response). The estimated odds ratio for fixation time was estimated to be 1.002 (95% CI 0.995–1.01).

#### Prolonged storage

To investigate the consequence of prolonged storage of tissue sections, sections of FFPE liver tissue, colon tissue, colon cancer tissue, and FFPE ABCG2 up-regulated cells were stored for 1 month at RT, 4 °C, or −20 °C or for 1 week at RT or 4 °C. Freshly cut sections left to dry ON were used as controls, and all sections were stained with the antibody BXP-21 (diluted 1:500 for tissue and 1:5000 for cell lines). Colon samples were assigned a score from 0–3 for basolateral membrane staining. Membrane staining of bile canaliculi was assessed in the liver, whereas both membrane and cytoplasmic staining were assessed in cell lines. In general, ABCG2 immunoreactivity was conserved in tissue samples as well as in paraffin-embedded cells stored at RT, 4 °C, or −20 °C for up to one month. Slight differences in the intensity of both membrane and cytoplasmic staining were observed in some specimens; however, there was no trend favoring any one storage approach (data not shown).

#### Tumor heterogeneity

**Figure 7:**
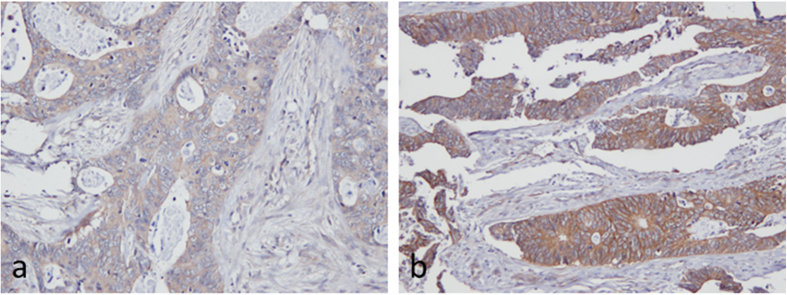
Heterogeneous expression of ABCG2 in a single CRC tissue sample. Immunostaining of ABCG2 in CRC tissue with BXP-21 (1:500 dilution) counterstained with Mayer’s hematoxylin, 10x magnification. (**a**) Low ABCG2 expression, scored as 1, in one area of the tumor. (**b**) High ABCG2 expression, scored as 3, in another area of the tumor.

Heterogeneous ABCG2 expression was observed in some whole sections of CRC tissue samples from the 72 mCRC patients, as shown in Fig. 7, in which tumor cells in part of the tumor exhibited strong basolateral membrane staining, whereas tumor cells in another part of same tumor displayed considerably less intense staining, thus clearly demonstrating that tumor heterogeneity is a concern in some tumors. The heterogeneity of ABCG2 immunoreactivity was examined in FFPE CRC TMAs and the corresponding whole sections to evaluate the representative value of TMAs in possible heterogenic CRC tumors. Four TMAs each with duplicate cores from 18 of the 72 patients were stained along with the corresponding whole sections. Whole sections were not available for eight patients, and therefore the corresponding TMA duplicates were not included in the analysis. Furthermore, seven core duplicates were lost during the cutting and staining protocols. Ultimately, 57 matching whole sections and TMAs were available for analysis. ABCG2 immunostaining in whole sections was assessed and given a score from 0 to 3 using the scoring guidelines described above. Core duplicates were evaluated in the same manner. In 14 cases, only one core was assessed because the other was lost during cutting and staining or because the core did not contain enough tumor cells (cores in duplicate, n = 44). The basolateral membrane scores from whole sections and corresponding TMAs were then compared. When duplicate scores were not identical, the maximum score was chosen (max TMA).

**Figure 8:**
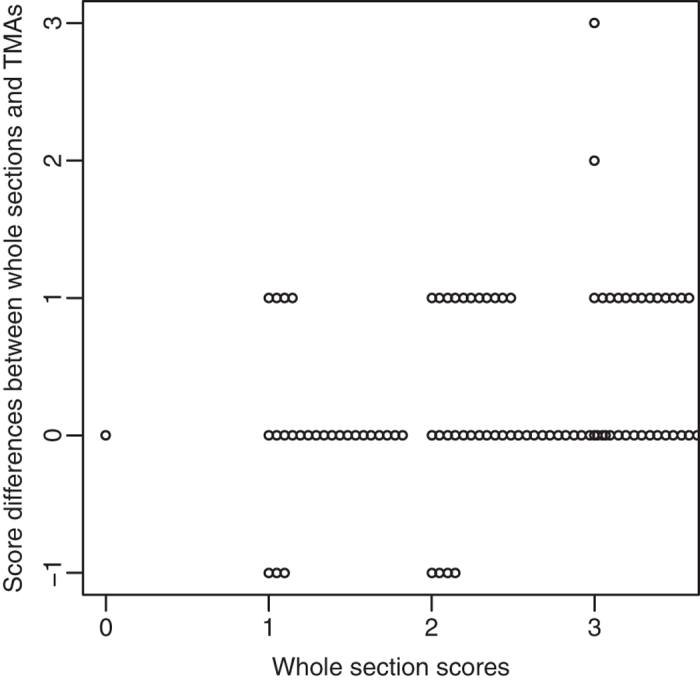
Difference between TMA scores and whole section scores. ABCG2 immunostaining in whole sections and TMAs was assessed, and the basolateral membranes were scored from 0 to 3 using the described scoring guidelines. For cases in which the duplicate TMA scores were not identical, the maximum score was chosen (max TMA). Most TMAs and whole sections were in concordance. In inconsistent cases, most TMAs were scored lower than whole sections resulting in differences in scores from 1–3.

The correspondence between TMAs and whole sections was relatively high, as shown in Fig. 8 and Supplementary Figure S9. Most discordant results differed by only one score, except for two TMAs with scores of 0 and 1 that had corresponding whole section scores of 3 (Fig. 8). The highest consistency was seen in TMAs with a score of 3 followed by TMAs with scores of 1 and 2. Finally, the lowest consistency was seen in TMAs with a score of 0. A Spearman’s correlation analysis was performed to determine the relationship between the 57 whole sections and the max TMA scores (both duplicates and single cores included). There was a strong positive correlation between max TMA score and whole sections (*r*_*s*_ = 0.71; 95% CI 0.56–0.82). A second Spearman’s correlation analysis was performed to determine the relationship between the 44 TMAs for which two cores were available (max TMA score used) and the corresponding whole sections. There was a strong positive correlation between max TMA score and whole sections (*r*_*s*_ = 0.82; 95% CI 0.71–0.89).

## Discussion

ABCG2 has been shown to be up-regulated in many cancers and to contribute to a multidrug resistance phenotype by transporting chemotherapeutic agents out of the cell. The correlation between ABCG2 expression and clinical outcome in CRC patients has been investigated with somewhat varying results^9^,^10^,^11^,^12^,^13^. Here, we provide the necessary analytical evidence for the use of mAb BXP-21 to detect and semi-quantitatively assess the ABCG2 protein in FFPE tumor tissue. With these validation data and the described scoring guidelines, it is now possible to study the predictive value of ABCG2 in tumor biopsies obtained from cancer patients.

Recommendations for proper antibody validation for IHC have been discussed and evaluated several times. In 2014, a consortium of academic and pharmaceutical-based histopathology researchers published their recommendations for antibody validation of immunohistochemistry for biomarker discovery^14^, including antibody testing in at least one non-IHC assay with proper positive and negative control cell lines. The validation procedure used in this study was modified from the “Rimm lab Algorithm for validation for IHC” suggesting two overall steps composed of WB- and IHC-based analyses^15^. Initially, the six antibodies were tested by WB, and then the specificity of the selected antibodies was further verified with ICC.

The mouse mAb BXP-21 detected a band at 72 kDa, corresponding to the expected size of ABCG2, in ABCG2 up-regulated cell lines. A clear down-regulation of this 72 kDa band was observed after ABCG2-specific siRNA transfection. By ICC, membrane staining was observed in all ABCG2 up-regulated cell lines and MCF7_DMSO_ in accordance with the WB results and previous studies^16^,^17^. Additional bands were observed for MDA-MB-231 and LoVo cells at approximately 250 kDa and at 15–20 kDa, respectively. However, these bands were still present after ABCG2-specific siRNA transfection, thus suggesting that these bands are derived from other proteins not related to ABCG2 and that they are not important for ICC, because no immunostaining was observed in MDA_DMSO_ and only weak immunostaining was detected in a small fraction of LoVo_parental_ cells. A weak 140–150 kDa band was detected for LoVo_SN-38RES_ and MDA_SN-38RES_ by WB but not for the parental cell lines. This band size corresponds with ABCG2 dimers, which have been described before by WB^18^. The observation of a complete disappearance of the band in WB after ABCG2-specific siRNA down-regulation of the ABCG2 protein makes this rationale probable. Another possibility is that the 140–150 kDa band represents cross-reaction to ABCB1, which is one of the major ABC transporters that has been shown to be expressed in normal intestine and to be up-regulated in CRC^19^. In the present study ABCB1 expression was not demonstrated in SN38 resistant MDA-MB-231 and MCF7 cell lines by WB, whereas an up-regulation of ABCB1 was detected for LoVo_SN-38RES_. Therefore, we examined BXP-21 in ABCB1 up-regulated cell lines. Several faint bands were observed by WB, including bands at 130–150 kDa in ABCB1 up-regulated cells as well as parental MDA-MB-231 and MCF7 cell lines. However, if BXP-21 cross-reacted with ABCB1, we would have expected a very intense band at 140–150 kDa in the ABCB1 up-regulated cell lines. No immunostaining of the membrane was observed in either MDA_parental_ or MDA_DTXRES_ by ICC, whereas membrane staining was demonstrated by ICC in the ABCB1 up-regulated and parental MCF7 cell lines because MCF7 expresses low levels of ABCG2. We would have expected stronger membrane staining in the MCF7 ABCB1 up-regulated cell line if there were a cross-reaction with ABCB1. Maliepaard and colleagues^4^ have previously tested the specificity of BXP-21 by WB in ABCG2-expressing cell lines and have observed no cross-reactivity with ABCB1, consistently with the results obtained in this study.

Nearly identical results to mAb BXP-21 were obtained with the mouse mAb 6D171 by WB, ICC and IHC. This was not surprising because BXP-21 and 6D171 were raised against peptides with identical amino acid sequences and may therefore recognize the same epitope.

The antibodies mAb 3G8, pAb TA324234 and pAb TA332085 all detected equally strong bands in ABCG2 up-regulated and parental cell lines, leading to disqualification of these antibodies. An ABCG2 splice variant at 67 kDa has been described, which is close to the band size of ~60 kDa demonstrated by mAb 3G8. However, the detection of equally strong bands in LoVo_SN-38RES_ and MDA_SN-38RES_ as well as LoVo_parental_ and MDA_DMSO_ made 3G8 ineligible for further analysis.

Bands at 72–75 kDa were detected with the pAb B7185 in both ABCG2 up-regulated and parental cell lines. Unexpectedly, the band observed in LoVo_parental_ was significantly more intense than in LoVo_SN-38RES_, in which two faint but distinct bands were observed in proximity. If the band was the ABCG2 protein, this would suggest a higher protein expression in LoVo_parental_, which does not correlate with our published data reporting higher levels of *ABCG2* mRNA in SN-38 resistant LoVo cells^20^. However, a slight decrease in intensity was observed in the upper band in ABCG2-specific siRNA-transfected LoVo_SN-38RES._ In agreement with the WB results, strong membrane staining was observed by ICC in ABCG2 up-regulated cells as well as parental cells. These results imply that B7185 does in fact react with ABCG2. However, the strong membrane staining demonstrated by ICC in parental cells and in siRNA down-regulated LoVo_SN-38RES_ and MDA_SN-38RES_ suggests that B7185 reacts with additional membrane proteins, consequently disqualifying B7185 for IHC of tissues.

Very short fixation and prolonged fixation might influence the immunostaining results of some biomarkers^21^; therefore, evaluation of these parameters is important when implementing new biomarker IHC protocols in clinical pathology laboratories. We therefore investigated the effects of fixation time on immunoreactivity, using the validated mouse mAb BXP-21. Analyses of FFPE LoVo_SN-38RES_ with different fixation times revealed no difference in ABCG2 staining in cells fixed for up to one month. In the 19 CRC samples, no statistical association between fixation time and basolateral membrane score was observed. This result strongly indicates that the ABCG2 antigen is robust in relation to different fixation times, which is a considerable advantage when performing studies including archived tumor material.

In clinical laboratories, several sections are often cut in one session to avoid excessive cutting of valuable tumor material, and the unused FFPE sections are stored. However, reduced immunoreactivity might occur when unstained FFPE sections are stored on glass slides for a prolonged amount of time. The reduction in immunoreactivity is antigen dependent. Interestingly, it has been found that immunoreactivity in nuclear and membrane antigens decreases over time, whereas cytoplasmic antigens maintain immunoreactivity for longer periods of time^22^. This may present a caveat for membranous staining of ABCG2. Furthermore, the antigen decay was observed to be light and temperature dependent^22^,^23^. However, in the present study, FFPE cells and tissue sections were stored under dark conditions for 1 month at RT, 4 °C, or −20 °C or for 1 week at RT or 4 °C, and there was no noticeable reduction observed in ABCG2 immunostaining under these conditions.

Few studies have analyzed ABCG2 expression in CRC tissue with IHC. Different assessment systems have been used because no standardized scoring system has been established. Diestra and colleagues have investigated ABCG2 expression in different cancers including CRC. In their work, staining was regarded as positive if >10% of tumor cells were stained, and they observed ABCG2 expression in CRC but did not differentiate between cytoplasmic and membrane staining^3^. Surprisingly, Gupta and colleagues have found decreased ABCG2 expression in CRC tissue compared to normal colon tissue, but did not account for staining assessment^24^. A more elaborate scoring system has been described in a study by Liu and colleagues. Here, the number of positive cells were scored as 0, <5%; 1, 5–25%; 2, 25–50%; 3, 50–75%; and 4, >75%. Additionally, intensity was scored as 0, negative; 1+, weak; 2+, moderate; and 3+, strong. Scores were multiplied for a final score in the range of 0–12. Scores of 0–4 were defined as ABCG2 low expression, whereas scores of 5–12 were defined as ABCG2 high expression. Positive staining was mainly found in membranes; however, cytoplasmic staining was included in the score^10^. A similar scoring system has been used by Wang and colleagues, who investigated the prognostic value of ABCG2 in CRC patients. The product of intensity (0–3) and the number of positive tumor cells (1–4) was translated into four final scores. These were defined as follows: 0, – (negative); 1–3, + (weakly positive); 4, ++ (moderately positive); and ≥5, +++ (strongly positive). Both membrane and cytoplasmic staining were evaluated separately; however, apical versus basolateral membrane staining was not considered. The authors have found that strong membranous ABCG2 expression is significantly associated with higher Dukes’ stage, lymph node metastasis, and distant metastasis and that it is an independent prognostic factor of overall survival^11^. In a more recent study by Silvestris and colleagues, the predictive role of ABCG2 expression has been examined in mCRC patients treated with a first-line FOLFIRI regimen^12^. Sections were scored as follows: 0 (no positive cells), 1 (≤10% positive cells), and 2 (>10% positive cells). Scores of 0 and 1 were defined as negative cells, and a score of 2 was used to define positive cells. Both cytoplasmic and membrane staining were included in the score, although most positive tumors exhibited membranous staining. No association between ABCG2 expression and clinical outcome (complete or partial response) was found. Another recent study by Trumpi and colleagues has found no correlation between ABCG2 expression in the primary tumor and the response of the corresponding metastases to irinotecan therapy in patients with metastatic CRC. They used a TMA with one 2 mm core, and apical membrane and cytoplasmic expression were scored as follows: − = no staining, + = weak staining, ++ = moderate staining and +++ = strong staining^13^.

In summary, diverse scoring systems have been applied to evaluate ABCG2 staining in CRC tissues, which might explain the contradictory results.

On the basis of our observations of ABCG2 immunoreactivity in the included CRC samples, we defined scoring guidelines for future studies to validate the predictive value of ABCG2 immunoreactivity in cancer tissue. In the present study, observations in BXP-21-stained FFPE CRC tissues revealed three distinguishable ABCG2 expression patterns: apical membrane, basolateral membrane and cytoplasmic. ABCG2 immunostaining of the apical membrane of epithelial cells was observed in normal colon tissue and in some CRC tumors without basolateral membrane staining. However, because pseudo-luminal staining in mucous tissue has been described to be caused by nonspecific staining for HER2^25^, we chose to disregard the staining of apical membranes in tumor cells. Similarly, cytoplasmic staining was disregarded; because ABCG2 is a membrane protein, it is more likely that membrane staining, as opposed to cytoplasmic staining, would represent the functional protein. Consequently, we will apply the established HER2 IHC scoring guidelines^26^ routinely used in gastric cancer in our future validation of ABCG2 immunostaining in CRC.

TMAs are practical, economically advantageous and enable the analysis of several tissue samples simultaneously. This study revealed intra-tumor heterogeneity of ABCG2 immunostaining in tissue samples from some CRC patients. Therefore, we investigated the correlation between ABCG2 basolateral membrane staining in TMAs and whole sections to determine whether TMAs are representative of whole sections. Statistical analysis indicated a strong positive correlation between TMAs and whole sections when both single and duplicate cores were included (n = 57), demonstrating that TMAs can replace whole sections in IHC analysis of ABCG2. In most cases of inconsistencies between TMAs and whole sections, TMAs scored lower than whole sections. The risk of false negative TMA results theoretically decreases when more cores are added to the analysis, because additional cores would cover larger areas of the whole section, thereby increasing the chance of removing a core from a high ABCG2-expressing location. Indeed, the correlation was even stronger when we compared whole sections with duplicate cores only (n = 44), thus demonstrating that two cores improve the concordance and accuracy of TMAs. In a study conducted by Lin and colleagues, the concordance rates of ER, PR and HER2 expression between TMAs and whole sections have been investigated^27^. In agreement with our findings, they have found that non-concordance rates between TMAs and whole sections are inversely related to the number of cores, because non-concordance rates become markedly lower as more cores (1–3 cores) were included in the analysis. Tumor size does not appear to affect the concordance between TMAs and whole sections, thus suggesting that the number of cores rather than core size improves concordance^27^.

## Conclusion

In conclusion, after validation of commercially available ABCG2 antibodies, we established scoring guidelines for semi-quantitative measurement of ABCG2 in FFPE CRC tissue, based on the clinically used guidelines for HER2 assessment in gastric cancer. With preclinical data demonstrating the functional involvement of ABCG2 in resistance to several anti-cancer drugs, studies should now be undertaken to clinically investigate ABCG2 as a predictive biomarker, including validation of the proposed scoring protocol. If ABCG2 can be used as a predictive marker, e.g., for irinotecan efficacy, this would enable tailored treatment for individual patients, which ultimately should result in greater success rates in chemotherapeutic treatment and should decrease unnecessary drug-induced adverse events in patients with no benefit from the treatment.

## Methods

### Anti-ABCG2 antibodies

Six commercially available anti-ABCG2 antibodies were purchased from five vendors. The mouse monoclonal antibody (mAb) BXP-21 was purchased from Abcam (Cambridge, UK), and the immunogen was a fusion protein composed of E. coli maltose binding protein and ABCG2 peptide (aa271-396). The mouse mAb 3G8 was purchased from Abnova (Taipei City, Taiwan), and the immunogen was a recombinant protein corresponding to human ABCG2 (aa153-360). The mouse mAb 6D171 was purchased from Santa Cruz Biotechnology (Dallas, Texas, USA), and the immunogen was ABCG2 of human origin (aa271-396). The rabbit polyclonal antibody (pAb) B7185 was purchased from Sigma-Aldrich (St. Louis, Missouri, USA), and the immunogen was a synthetic peptide corresponding to aa150-167 of ABCG2 with an added C-terminal cysteine conjugated to keyhole limpet hemocyanin (KLH). The rabbit pAb TA324234 was purchased from Origene (Rockville, USA), and the immunogen was a synthetic peptide directed toward the N-terminal of human ABCG2 within the region aa50-99. The rabbit pAb TA332085 was also purchased from Origene, and the immunogen was a synthetic peptide corresponding to a region derived from aa609-621 of human ABCG2. The pAb TA324234 was the only antibody with an extracellular immunogen, whereas the immunogens for all of the other antibodies were from intracellular domains of ABCG2.

### Cell lines

The human CRC cell line LoVo and the human breast cancer cell line MDA-MB-231 were obtained from the American Type Culture Collection (ATCC, Rockville, MD), whereas the human MCF-7 breast cancer cell line was kindly provided by Professor Ole William Petersen (University of Copenhagen). For each of these cell lines, we have previously established SN-38 resistant sublines with acquired resistance by exposure to gradually increasing SN-38 concentrations^20^,^28^. An overview of the cell lines is shown in Table 1. The cell line identity of both parental and resistant cell lines was confirmed by Short Tandem Repeat (STR) DNA analysis (Identi Cell, Aarhus, Denmark). In addition, all cell lines were confirmed to be mycoplasma-free (Mycoplasma PCR Detection Kit, Minerva Biolabs, Berlin, Germany). These cell lines were chosen for the validation process of the anti-ABCG2 antibodies because the SN-38 resistant counterparts have been shown to over-express ABCG2, whereas their parental counterparts express little or no ABCG2. Furthermore, the functional importance of over-expressed ABCG2 has been demonstrated because inhibition of ABCG2 by the specific ABCG2 inhibitor Ko143 (Sigma Aldrich) restores the sensitivity to SN-38 in the resistant cell lines^20^,^28^. The parental controls for the MDA-MB-231 and MCF7 cell lines were grown under the same medium conditions as the resistant cell lines and were further supplemented with dimethyl sulfoxide (DMSO) because SN-38 was diluted in DMSO. The cell lines are referred to as LoVo_parental_, LoVo_SN-38RES_, MDA_DMSO_, MDA_SN-38RES_, MCF7_DMSO_, and MCF7_SN-38RES_.

In addition, docetaxel resistant MDA-MB-231 and MCF7 cells^29^ and their parental counterparts were used to evaluate potential cross-reactivity with the ABCB1 (P-glycoprotein (PGP), MDR1) protein. Resistant sublines were obtained by passaging cells in increasing concentrations of docetaxel and were maintained in docetaxel-supplemented medium. These cell lines are referred to as MDA_parental_, MDA_DTXRES_, MCF7_parental_, and MCF7_DTXRES_.

LoVo cells were maintained in Roswell Park Memorial Institute (RPMI) 1640 (1x) GlutaMAX^TM^ (Life Technologies) supplemented with 10% fetal bovine serum (FBS) (Sigma-Aldrich). MDA-MB-231 and MCF7 were maintained in Dulbecco’s Modified Eagle’s Medium (DMEM) (1x) GlutaMAX^TM^ (Life Technologies) supplemented with 10% FBS and 5% FBS + non-essential amino acids (Life Technologies), respectively. All cell lines were incubated at 37 °C in 5% CO_2_ and grown until 70–85% confluent.

### RNA interference

Knockdown of ABCG2 was performed in LoVo_parental_, LoVo_SN-38RES_, MDA_DMSO_ and MDA_SN-38RES_ cells. Three Sigma-Aldrich top ranked siRNAs as well as a universal negative control conjugated to 6-carboxyfluorescein were used. The siRNA sequences were as follows: 5′-GUCAACUCCUCCUUCUACA-3′ and antisense 5′-UGUAGAAGGAGGAGUUGAC-3′, 5′-GUCUAAGCAGGGACGAACA-3′ and antisense 5′-UGUUCGUCCCUGCUUAGAC-3′, and 5′-GGUUAUCACUGUGAGGCCU-3′ and antisense 5′-AGGCCUCACAGUGAUAACC-3′. The three siRNA target sequences were blasted against the human genomic plus transcript in the NCBI/blast online tool, (https://blast.ncbi.nlm.nih.gov/Blast.cgi?PAGE_TYPE=BlastSearch), and the target sequences were only identified in the ABCG2 mRNA. Cell lines were seeded in T75 culture flasks and 6-well plates 24 hours prior to transfection. LoVo_parental_ and LoVo_SN-38RES_ were seeded at a density of 4 × 10^5^ cells per well, MDA_DMSO_ was seeded at a density of 6 × 10^4^ cells per well, and MDA_SN-38RES_ was seeded at a density of 8 × 10^4^ cells per well. At 30–50% confluence, the medium was changed for a total volume of 8 mL in T75 flasks and 0.8 mL in wells. The siRNAs were pooled and diluted in Opti-MEM (Life Technologies) to obtain a concentration of 500 nM. The negative siRNA control was diluted separately. A mixture of Lipofectamine 2000 (Life Technologies) and Opti-MEM were incubated at room temperature (RT) for 5 minutes. Then, LF2000-Opti-MEM and siRNAs were mixed to a concentration of 250 nM and incubated at RT for 20 minutes. The LF2000-siRNA solutions were added to flasks and wells to final concentrations of 50 nM. Cells were allowed to incubate at 37 °C for 4–6 hours before substitution of LF2000-siRNA-containing medium with normal growth medium. Cells were harvested after 96 hours.

The siRNA transfection protocol was optimized, and each antibody (BXP-21, 6D171, and B7185) was tested twice by both WB and ICC.

### Protein extraction and immunoblotting

Protein extraction was performed with M-PER Mammalian Protein Extraction Reagent supplemented with Pierce™ Phosphatase Inhibitor Mini Tablets (Thermo Scientific, Life Technologies) according to the manufacturer’s protocol.

Lysates were mixed with MilliQ and 4x Laemmli sample buffer supplemented with dithiothreitol (DTT) to obtain samples containing 20 μg of total protein. Total protein was measured with a Pierce^TM^ BCA Protein Assay Kit (Thermo Scientific) according to manufacturer’s protocol prior to mixing. An ABCG2 transient overexpression lysate from HEK293T cells was purchased from Origene and diluted in MilliQ and 4x Laemmli sample buffer supplemented with DTT for 2 μg of total protein. Samples were heated at 70 °C for 10 minutes, and equal amounts (20 μg) were loaded to Mini-PROTEAN® TGX^TM^ Gels (Bio-Rad Laboratories, Hercules, CA, USA) and run at 160–170 V for 40–45 minutes in running buffer consisting of Tris/Glycine/SDS buffer (Bio-Rad Laboratories). Proteins were transferred to 0.2 μm nitrocellulose Trans-Blot^®^ Turbo^TM^ Transfer Packs (Bio-Rad Laboratories) at 25 V for 10 minutes. Membranes were blocked for one hour at RT with either 5% skim milk or 5% BSA dissolved in Tris-buffered saline (TBS) wash buffer (TBS: 0.05 M Tris and 0.15 M NaCl, pH 7.6; TBS wash buffer: TBS+0.05% Tween) depending on the antibody. Next, the membranes were incubated with primary antibody diluted in blocking solution overnight (ON) at 4 °C with gentle agitation: anti-ABCG2 (clone BXP-21, Abcam) 1:1000, anti-ABCG2 (clone 3G8, Abnova) 1:500, anti-ABCG2 (clone 6D171, Santa Cruz Biotechnologies) 1:1000, anti-ABCG2 (clone B7185, Sigma-Aldrich) 1:5000, anti-ABCG2 (clone TA332085, Origene) 1:2000, anti-ABCG2 (clone TA324234, Origene) 1:1000, anti-ABCB1 (clone EPR10364-57, Abcam) 1:1000, anti-P150 (BD Biosciences, San Jose, CA, USA) 1:4000, and anti- β-actin (Sigma-Aldrich) 1:200,000. Subsequently, the membranes were incubated with secondary anti-mouse or anti-rabbit antibodies (Dako, Glostrup, Denmark) diluted 1:4000 in 5% skim milk or 5% BSA and Precision Protein™ StrepTactin-HRP Conjugate (Bio-Rad Laboratories) diluted 1:50,000 for 1 hour at RT with gentle agitation. Solutions of luminol and peroxide in a 1:1 ratio (Clarity^TM^ Western ECL, Bio-Rad Laboratories or Amersham ECL Select WB Detection Reagent, GE Healthcare Life Sciences, Buckinghamshire, UK) were prepared and applied to the membranes, and the membranes were incubated at RT for 5 minutes in the dark. Protein bands were visualized using the UVP BioSpectrum Imaging System. For re-probing with β-actin and ABCB1 antibodies, the membranes were washed with TBS wash buffer and stripped with Restore^TM^ PLUS WB Stripping Buffer (Life Technologies).

### Paraffin embedding of cell lines

At 70–85% confluence, the cells were washed twice with cold phosphate buffered saline (PBS) (Life Technologies) and fixed in 10% neutral buffered formalin (NBF) for 30 minutes. Formalin was discarded, and the cells were washed twice with PBS and detached with a cell scraper. The cell suspension was centrifuged for 10 minutes at 400 G at 4 °C. The pellet was re-suspended in 10–20 μL PBS and 25 μL of the cell suspension was injected into a drop of warm liquid 2% bactoagar on a glass slide. Droplets were allowed to cool at 4 °C and transferred to a cassette. The cell agar droplets were kept in 70% ethanol and embedded in paraffin after dehydration in rising concentrations of ethanol and xylene. To investigate the influence of different fixation times, LoVo_parental_ and LoVo_SN-38RES_ were fixed in 10% NBF for 5 minutes, 30 minutes, 6 hours, 1 week or 1 month.

### Tissue

Primary tumors from 9 untreated CRC patients, normal colon tissues and liver tissues fixed in 10% NBF were obtained from Herlev and Hvidovre Hospitals.

Fixation times: Fresh tumor tissue from 19 untreated CRC patients who had undergone surgery at Odense University Hospital was trimmed for normal intestinal tissue and cut into approximately 2-mm-thick slices. Tissue sections were fixed in 10% NBF for 3 to 288 hours. Subsequently, tissue samples were embedded in paraffin^30^.

Heterogeneity of ABCG2 in colon cancer: Primary tumors from 72 CRC patients, who initial had or later developed metastatic disease (mCRC), were extracted from a Danish national cohort of 498 patients with mCRC who had all received irinotecan in combination with the epidermal growth factor receptor inhibitor cetuximab as a third-line treatment from January 1^st^, 2005 to August 1^st^, 2008 at the Departments of Oncology at Herlev, Odense, and Aalborg Hospitals in Denmark. The 72 patients included in this study were randomly selected, thus representing a heterogeneous group. TMA blocks were produced at Herlev Hospital; each contained tumor material from 18 patients with two 1 mm tissue cores per patient sample. Standard procedures were used for preparation of the TMA blocks. TMA blocks were compared with the corresponding whole sections.

The study was conducted in accordance with the ethical standards of the 1964 Helsinki declaration and its later amendments. The use of tumor tissue from mCRC patients was approved by the Research Ethics Committee of Copenhagen (H-KA-20060094).

For this type of study, formal consent is not required because no identifying information relating to participants was included.

### Immunocytochemistry and immunohistochemistry

Immunostaining was performed on 3 μm paraffin sections mounted on SuperFrost plus slides (Thermo Scientific). Sections were deparaffinized in xylene and re-hydrated in a graded series of ethanol. Epitope retrieval was achieved by boiling sections in Target retrieval solution at pH 6 or pH 9 (Dako) in a domestic microwave for 5 or 10 minutes, after which sections were allowed to cool in retrieval solution for 20 minutes at RT. For the antibody clone BXP-21, the best result was obtained with 5 minutes of boiling in Target retrieval solution at pH 9. Epitope retrieval was followed by blocking for 10 minutes in 1% peroxide solution and washing with TBS+0.5% Triton X-100 prior to incubation with primary antibody. A titration of antibodies was performed, and the optimal results for ICC were obtained with dilutions of 1:3000 for clone BXP-21 and clone 6D171, 1:500 for clone B7185, and 1:1000 for the anti-ABCB1 (clone EPR10364-57, Abcam). For IHC on normal tissue, the optimal dilutions were 1:500 for clone BXP-21 and clone 6D171 and 1:400 for clone B7185. The antibodies were diluted in Ready-to-use Antibody Diluent with background reducing agents (Dako) for 1 hour at RT. Subsequently, sections were incubated with HiDef Detection^TM^ Amplifier (Cell Marque) for 20 minutes and with HiDef Detection^TM^ Polymer Detector (Cell Marque) for 20 minutes at RT. Sections were washed in TBS+0.5% Triton X-100 between incubations. A final wash with TBS was performed before the signal was visualized with the chromogen 3’3-Diaminobenzidine (DAB+) (Dako) for 10 minutes.

### Prolonged storage of whole sections

Sections from FFPE normal liver and colon tissue, CRC tissue as well as paraffin-embedded LoVo_parental_, LoVo_SN-38RES_, MDA MB231 _DMSO_, MDA MB231 _SN-38RES_, MCF7_DMSO_, and MCF7_SN-38RES_ cell lines were used to determine whether prolonged storage at RT, 4 °C, and −20 °C affected the immunoreactivity of ABCG2 detected by IHC. Sections were cut into 3 μm sections. Part of the sections were stored for 1 month at RT, 4 °C, or −20 °C, and part of the sections were stored for 1 week at RT or 4 °C in plastic slide boxes to prevent exposure to light. Fresh sections were cut the day before staining and were allowed to dry at RT ON.

### Statistics

A proportional odds model was used to analyze the effect of fixation (exposure) on the score (response).

Spearman’s correlation test was performed to investigate the heterogeneity in the CRC samples.

The statistical software R version 3.1.3 was used^31^.

## Additional Information

**How to cite this article**: Cederbye, C. N. *et al.* Antibody validation and scoring guidelines for ABCG2 immunohistochemical staining in formalin-fixed paraffin-embedded colon cancer tissue. *Sci. Rep.* **6**, 26997; doi: 10.1038/srep26997 (2016).

## Supplementary Material

Supplementary Information

## Figures and Tables

**Table 1:** Cell line overview.

LCell lines	Treatment	Up-regulated protein	Referred to as
LoVo	None	–	LoVo_parental_
	SN-38	ABCG2	LoVo_SN-38RES_
MDA-MB-231	DMSO	–	MDA_DMSO_
	SN-38	ABCG2	MDA_SN-38RES_
	None	–	MDA_parental_
	Docetaxel	ABCB1	MDA_DTXRES_
MCF7	DMSO	–	MCF7_DMSO_
	SN-38	ABCG2	MCF7_SN-38RES_
	None	–	MCF7_parental_
	Docetaxel	ABCB1	MCF7_DTXRES_

**Table 2:** Outline of IHC scoring guidelines.

Score	Staining pattern	Membrane staining intensity	Magnification
**0**	No basolateral membrane staining or basolateral membrane staining in less than 10% of the tumor cells	No staining/barely visible	Use 40x objective
**1**	Weak basolateral membrane staining in ≥10% tumor cells	Barely visible	Use 40x objective
**2**	Weak to moderate basolateral membrane staining in ≥10% tumor cells	Weak to moderate	Use 10x–20x objective
**3**	Strong basolateral membrane staining in ≥10% tumor cells	Strong	Use 2.5x–5x objective
