# Supplementary material for: Antibody validation and scoring guidelines for ABCG2 immunohistochemical staining in formalin-fixed paraffin-embedded colon cancer tissue
Source: Sci Rep. 2016 Jun 3;6:26997. doi: 10.1038/srep26997 (PMC4891695; doi:10.1038/srep26997)
Supplement: Supplementary Information [file srep26997-s1.pdf]

Supplementary Information

Antibody validation and scoring guidelines for ABCG2 immunohistochemical staining in formalin-fixed paraffin embedded colon cancer tissue

Camilla Natasha Cederbye[1], Jesper Andreas Palshof[2], Tine Plato Hansen[3], Anne Katrine Duun-Henriksen[4], Dorte Linnemann[5], Jan Stenvang[1], Dorte Lisbet Nielsen[2], Nils Brünner[1], and Birgitte Martine Viuff[1]

Affiliations
1. Faculty of Health and Medical Sciences, Department of Veterinary Disease Biology, Section for Molecular Disease Biology, University of Copenhagen, Strandboulevarden 49, DK- 2100 Copenhagen Ø, Denmark
2. Department of Oncology, Herlev and Gentofte Hospital, University of Copenhagen, Herlev Ringvej 75, DK-2730 Herlev, Denmark
3. Department of Pathology, Hvidovre Hospital, Kettegaard Alle 30, DK- 2650 Hvidovre, Denmark
4. Danish Cancer Society, Strandboulevarden 49, DK- 2100 Copenhagen Ø, Denmark
5. Department of Pathology, Herlev and Gentofte Hospital, University of Copenhagen, Herlev Ringvej 75, DK-2730 Herlev, Denmark

Correspondence:
Birgitte Martine Viuff
Faculty of Health and Medical Sciences, Department of Veterinary Disease Biology, Section for Molecular Disease Biology, University of Copenhagen, Strandboulevarden 49, DK- 2100 Copenhagen Ø, Denmark
Email: bmv@sund.ku.dk

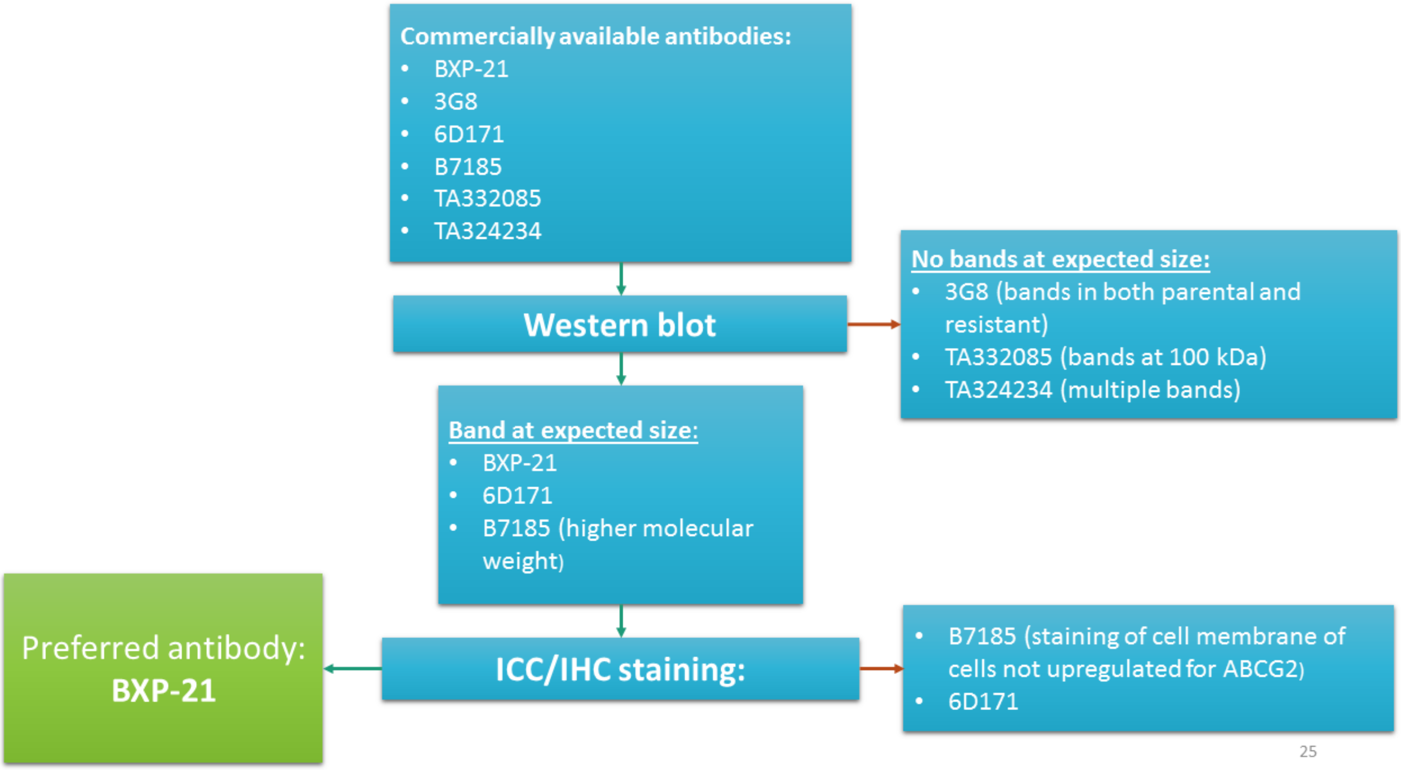

Supplementary figure S1 Flowchart illustrating the validation process of six commercially available anti-ABCG2 antibodies. Three antibodies were excluded based on western blot results. Three antibodies were examined by ICC and IHC. The mouse mAb anti-ABCG2 antibody BXP-21 was used in the subsequent IHC analyses of clinical samples.

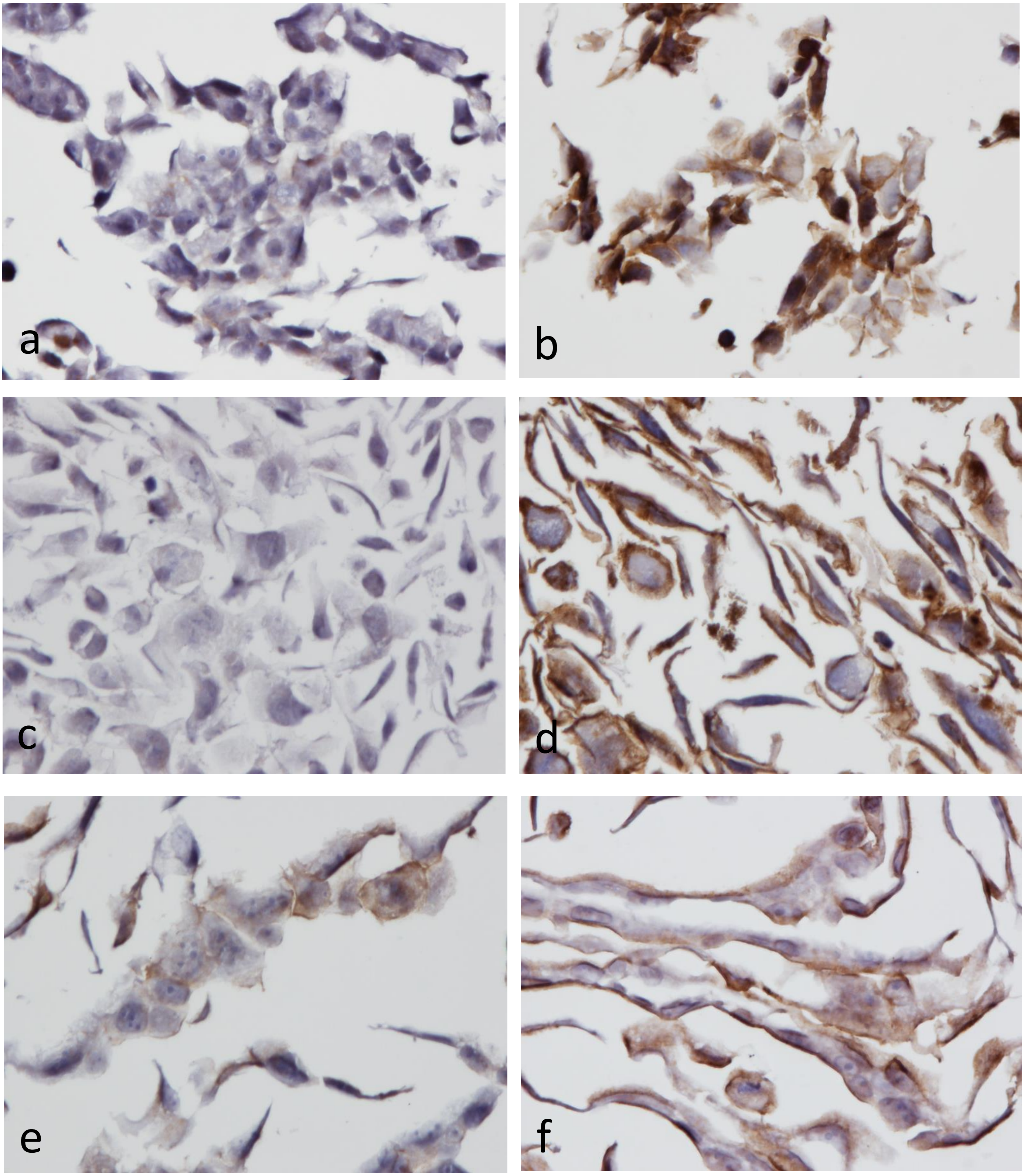

Supplementary figure S2 Immunostaining of ABCG2 in LoVo, MDA-MB-231, and MCF7 $_{\text{parental}}$ and ABCG2 up-regulated cell lines using mAb 6D171 (1:3000 dilution). ABCG2 was visualized with DAB, and sections were counterstained with Mayer’s hematoxylin, 40x magnification. (a) LoVo $_{\text{parental}}$ (b) LoVo $_{\text{SN-38RES}}$ (c) MDA $_{\text{DMSO}}$ (d) MDA $_{\text{SN-38RES}}$ (e) MCF7 $_{\text{DMSO}}$ and (f) MCF7 $_{\text{SN-38RES}}$.

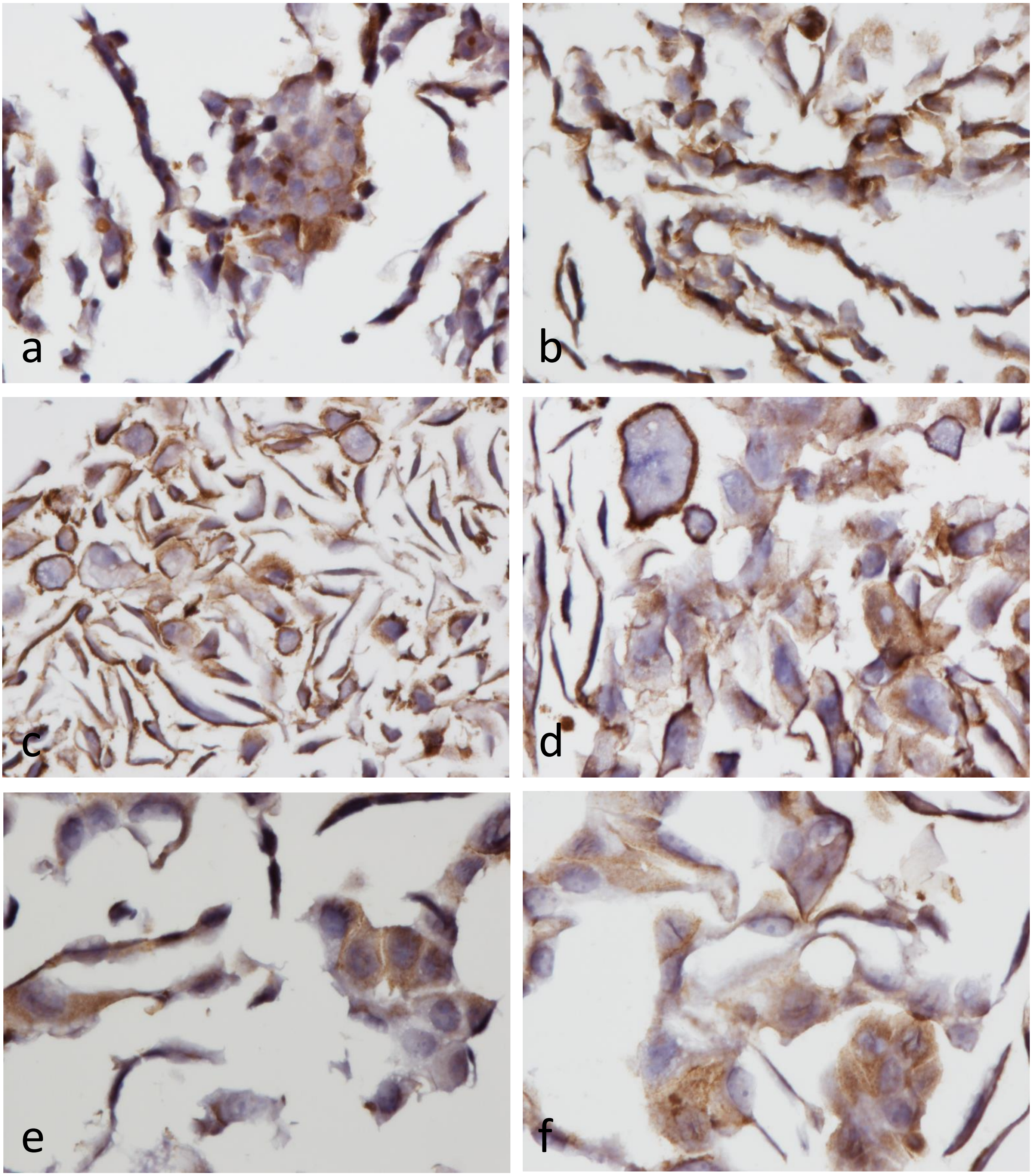

Supplementary figure S3 Immunostaining of ABCG2 using pAb B7185 (1:500 dilution) on LoVo, MDA-MB-231, and MCF7 $_{\text{parental}}$ and ABCG2 up-regulated cell lines. ABCG2 was visualized with DAB (brown) and sections were counterstained with Mayer's hematoxylin, 40x magnification. (a) LoVo $_{\text{parental}}$ (b) LoVo $_{\text{SN-38RES}}$ (c) MDA $_{\text{DMSO}}$ (d) MDA $_{\text{SN-38RES}}$ (e) MCF7 $_{\text{DMSO}}$ and (f) MCF7 $_{\text{SN-38RES}}$.

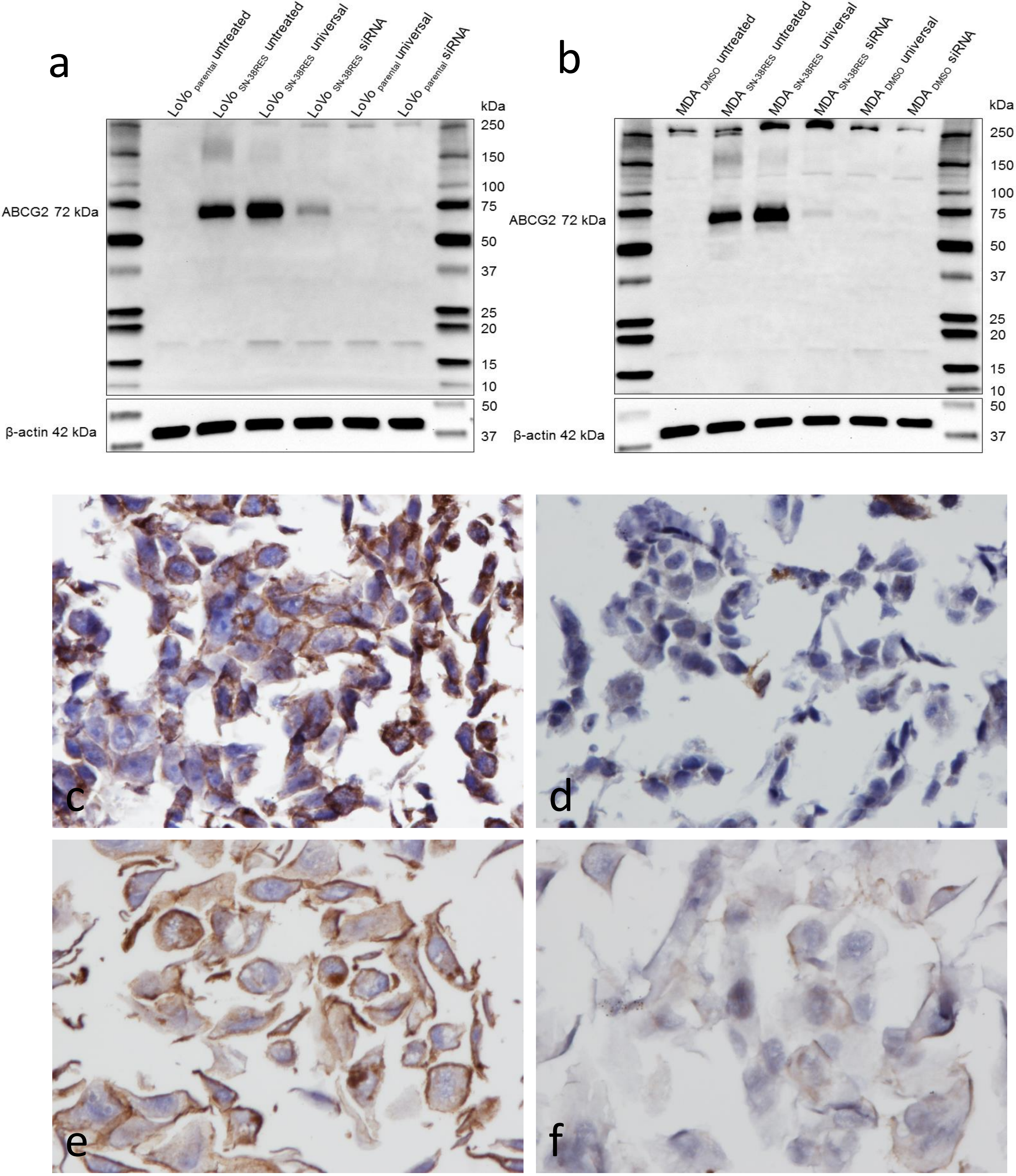

Supplementary figure S4

Supplementary figure S4 a-b: Western blot evaluation of mAb 6D171 specificity by siRNA-mediated down-regulation of ABCG2 (72 kDa). Two parental cell lines (LoVo $_{parental}$ and MDA $_{DMSO}$) were analyzed alongside their SN38 resistant counterparts (LoVo $_{SN\text{-}38RES}$ and MDA $_{SN\text{-}38RES}$) with up-regulated levels of ABCG2 as indicated above the membranes. (a) LoVo $_{parental}$ and LoVo $_{SN\text{-}38RES}$ and (b) MDA $_{DMSO}$ and MDA $_{SN\text{-}38RES}$. The cells were untreated, transfected with a universal negative control siRNA (universal) or transfected with a mixture of three different ABCG2-targeting siRNAs as indicated above the membranes. β-actin (42 kDa) was used as a loading control. The molecular weight marker is indicated.

c-f: Immunostaining of ABCG2 in siRNA down-regulated LoVo $_{SN\text{-}38RES}$ and MDA $_{SN\text{-}38RES}$ with mAb 6D171 (1:3000 dilution). ABCG2 was detected using the HiDef Detection^{TM} HRP Polymer system and counterstained with Mayer's hematoxylin, 40x magnification. (c) LoVo $_{SN\text{-}38RES}$ transfected with universal siRNA. (d) LoVo $_{SN\text{-}38RES}$ transfected with ABCG2-specific siRNA. (e) MDA $_{SN\text{-}38RES}$ transfected with universal siRNA. (f) MDA $_{SN\text{-}38RES}$ transfected with ABCG2-specific siRNA. The results are similar to the results seen for BXP-21 with a partial ABCG2 down-regulation in LoVo $_{SN\text{-}38RES}$ and an almost complete ABCG2 down-regulation in MDA SN-38.

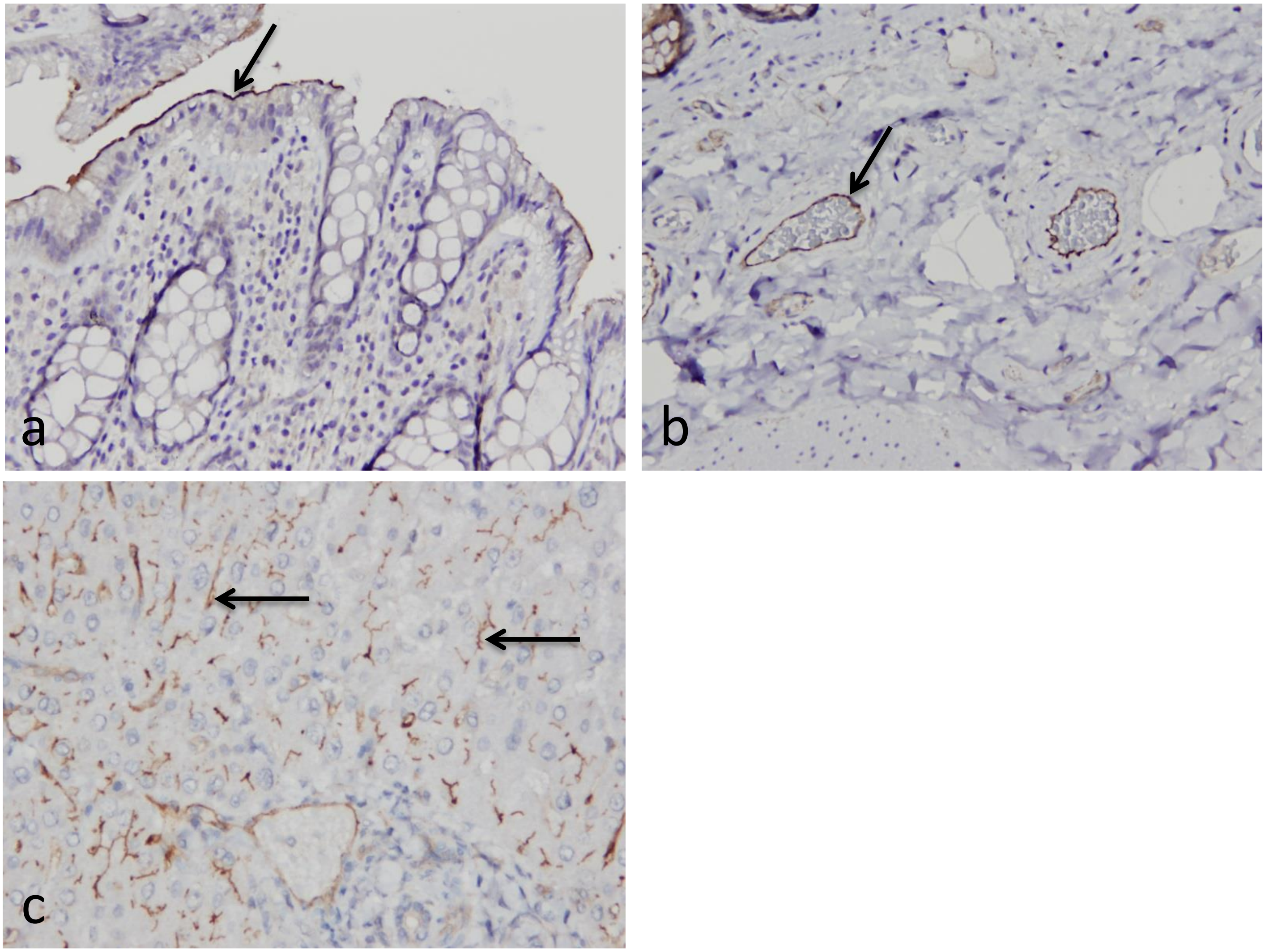

Supplementary figure S5 Immunostaining of ABCG2 in normal tissue using BXP-21. ABCG2 was visualized with DAB+ and counterstained with Mayer's hematoxylin, 20x magnification (a) Normal colon tissue with distinct staining of the apical membrane of epithelial cells, exemplified by the arrow (b) Normal colon tissue with distinct staining of endothelial cells in the lamina propria, exemplified by the arrow (c) Liver tissue with distinct staining of bile canaliculi, exemplified by arrows.

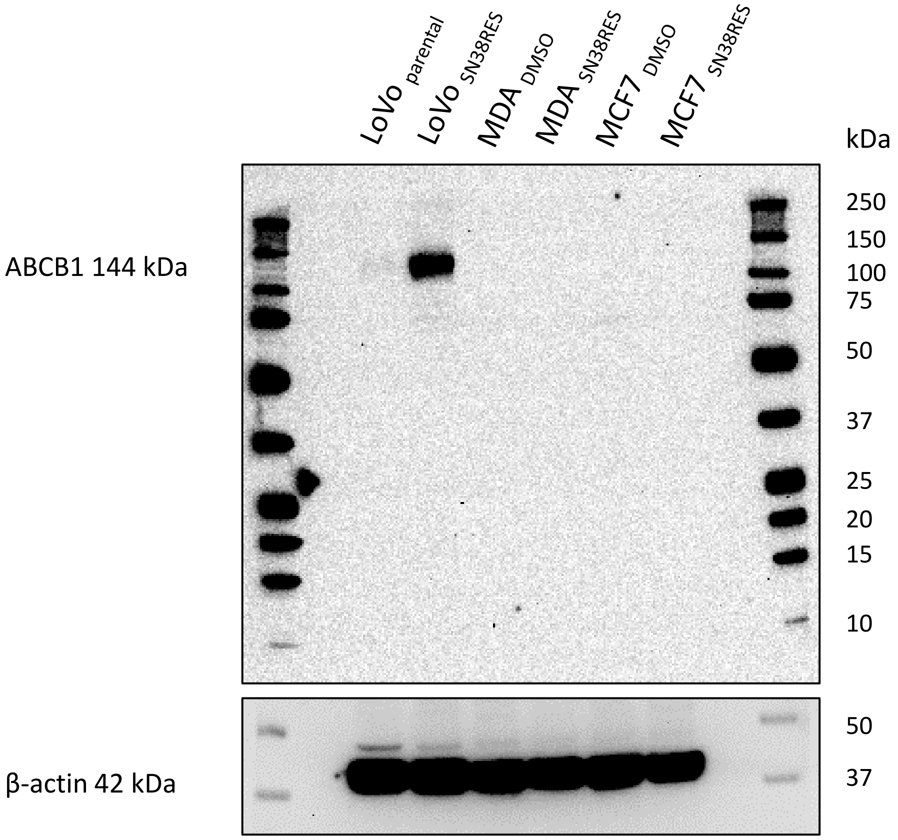

Supplementary figure S6 Western blot evaluation of ABCB1 expression in ABCG2 up-regulated cell lines. The three SN38 resistant cell lines (LoVo $_{SN\text{-}38RES}$, MDA $_{SN\text{-}38RES}$, and MCF7 $_{SN38RES}$) with up-regulated levels of ABCG2 were analyzed alongside their parental counterparts (LoVo $_{parental}$, MDA $_{DMSO}$, and MCF7 $_{DMSO}$) as indicated above the membranes. The anti-ABCB1 mAb (clone EPR10364-57, Abcam) was used to demonstrate ABCB1. β-actin (42 kDa) was used as a loading control. The molecular weight marker is indicated. Only the SN38 resistant cell line, LoVo $_{SN\text{-}38RES}$ expressed detectable levels of ABCB1.

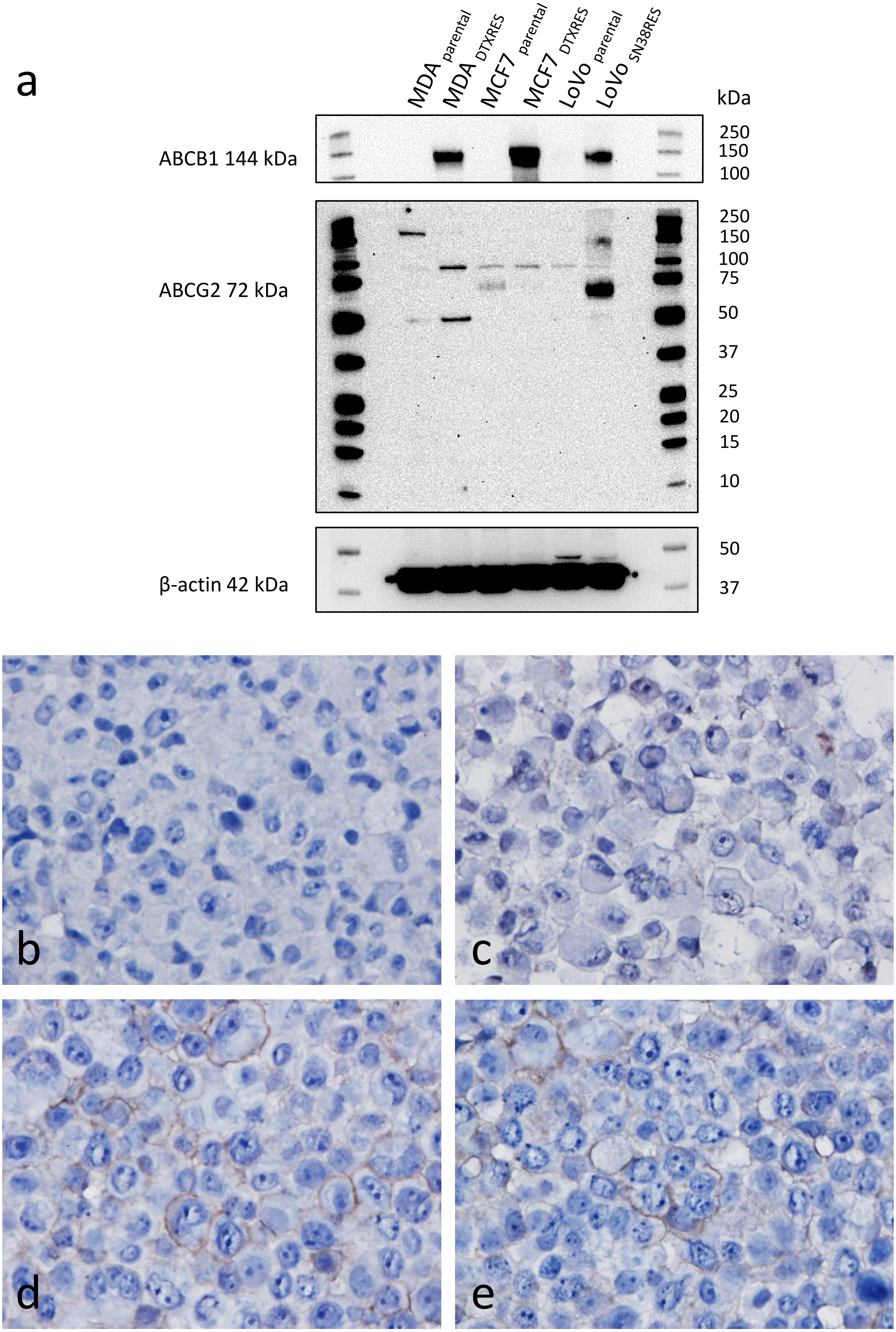

Supplementary figure S7

Supplementary figure S7 a: Western blot evaluation of mAb BXP-21 potential cross-reactivity toward ABCB1 (predicted molecular weight of 144 kDa). Two parental cell lines (MDA $_{parental}$ and MCF7 $_{parental}$) were analyzed alongside their docetaxel-resistant counterparts (MDA $_{DTXRES}$ and MCF7 $_{DTXRES}$) with up-regulated levels of ABCB1 as indicated above the membrane. Firstly, mAb BXP-21 was evaluated and for this purpose the full membrane is displayed to reveal the presence of all reactive bands upon antibody incubation. Secondly, the membrane was stripped and the upper part of the membrane was re-probed with anti-ABCB1 (144 kDa) whereas the lower part of the membrane was re-probed with anti-β-actin (42 kDa) which was used as a loading control. The molecular weight marker is indicated.

b-e: Immunostaining of ABCG2 in parental MDA and MCF7 cell lines and their docetaxel-resistant counterparts with BXP-21 (1:3000 dilution). ABCG2 was detected using the HiDef Detection^{TM} HRP Polymer system and counterstained with Mayer's hematoxylin, 40x magnification. (b) MDA $_{parental}$ (c) MDA $_{DTXRES}$ (d) MCF7 $_{parental}$ and (e) MCF7 $_{DTXRES}$. No membrane staining was observed in the ABCB1 up-regulated cell line MDA $_{DTXRES}$. The membrane staining detected in MCF7 $_{DTXRES}$ probably represents ABCG2 as the intensity of the immunoreaction was the same for MCF7 $_{parental}$ and MCF7 $_{DTXRES}$.

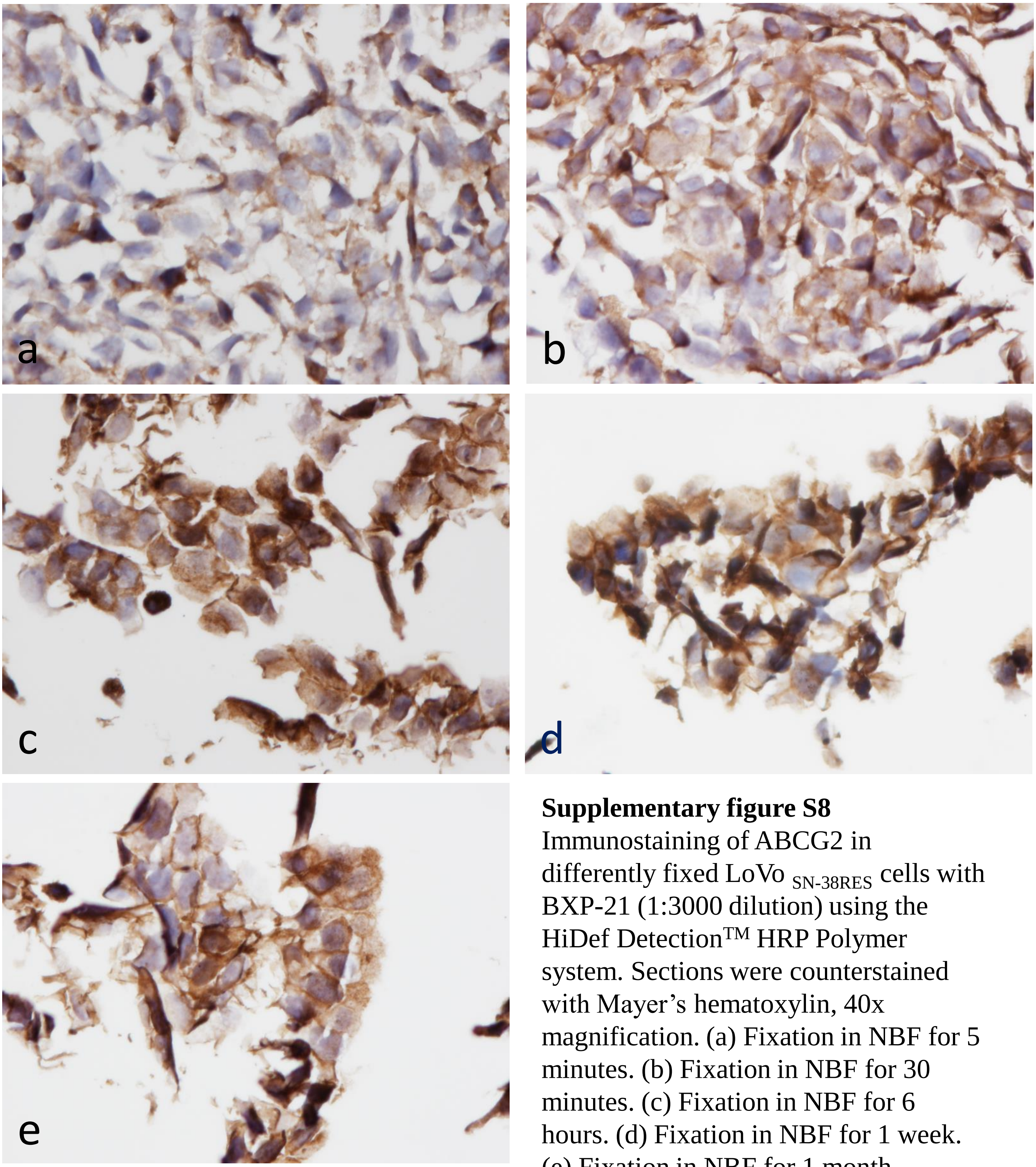

Supplementary figure S8
Immunostaining of ABCG2 in differently fixed LoVo $_{SN\text{-}38RES}$ cells with BXP-21 (1:3000 dilution) using the HiDef Detection^{TM} HRP Polymer system. Sections were counterstained with Mayer's hematoxylin, 40x magnification. (a) Fixation in NBF for 5 minutes. (b) Fixation in NBF for 30 minutes. (c) Fixation in NBF for 6 hours. (d) Fixation in NBF for 1 week. (e) Fixation in NBF for 1 month.

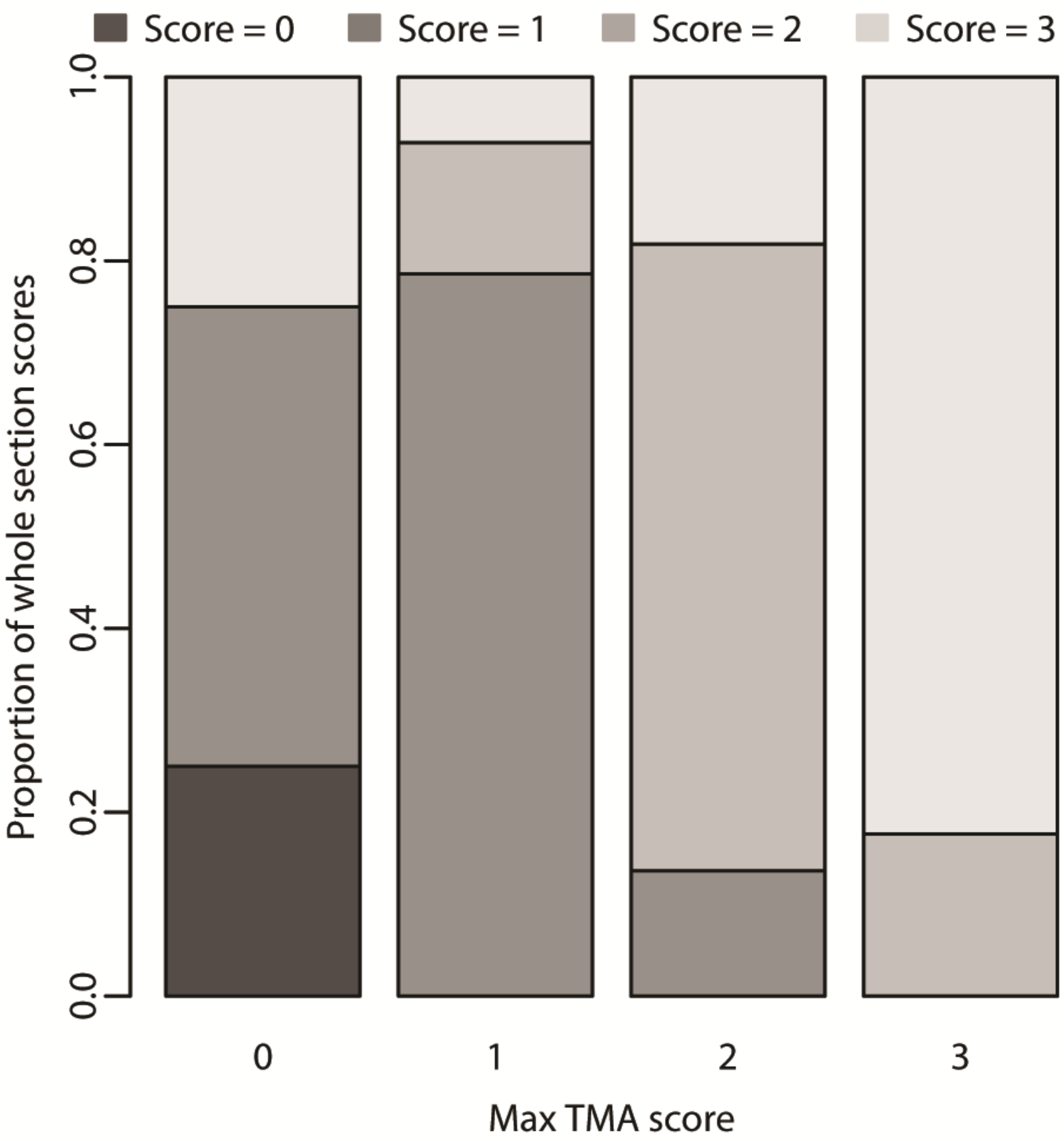

Supplementary figure S9 Concordance between whole sections (X-axis) and the max TMA scores (Y-axis). ABCG2 immunostaining in whole sections and TMAs was assessed, and the basolateral membranes were scored from 0 to 3 using the described scoring guidelines. For cases in which the duplicate TMA scores were not identical, the maximum score was chosen (max TMA). The bar diagram shows a high degree of concordance between whole sections and TMAs (n = 57).
